# Enzymatic synthesis and phosphorolysis of 4(2)-thioxo- and 6(5)-azapyrimidine nucleosides by *E. coli* nucleoside phosphorylases

**DOI:** 10.3762/bjoc.12.254

**Published:** 2016-12-01

**Authors:** Vladimir A Stepchenko, Anatoly I Miroshnikov, Frank Seela, Igor A Mikhailopulo

**Affiliations:** 1 Institute of Bioorganic Chemistry, National Academy of Sciences, Acad. Kuprevicha 5/2, 220141 Minsk, Belarus; 2 Shemyakin and Ovchinnikov Institute of Bioorganic Chemistry, Russian Academy of Sciences, Miklukho-Maklaya 16/10, 117997 GSP, Moscow B-437, Russia; 3 Laboratory of Bioorganic Chemistry and Chemical Biology, Center for Nanotechnology, Heisenbergstraße 11, D-48149 Münster, Germany

**Keywords:** enzymatic glycosylation, PM3 and ab initio calculations, recombinant *E. coli* uridine, thymidine and purine nucleoside phosphorylases, substrate properties, 4(2)-thioxo- and 6(5)-aza-uacil and -thymine

## Abstract

The *trans*-2-deoxyribosylation of 4-thiouracil (^4S^Ura) and 2-thiouracil (^2S^Ura), as well as 6-azauracil, 6-azathymine and 6-aza-2-thiothymine was studied using dG and *E. coli* purine nucleoside phosphorylase (PNP) for the in situ generation of 2-deoxy-α-D-ribofuranose-1-phosphate (dRib-1P) followed by its coupling with the bases catalyzed by either *E. coli* thymidine (TP) or uridine (UP) phosphorylases. ^4S^Ura revealed satisfactory substrate activity for UP and, unexpectedly, complete inertness for TP; no formation of 2’-deoxy-2-thiouridine (^2S^Ud) was observed under analogous reaction conditions in the presence of UP and TP. On the contrary, ^2S^U, ^2S^Ud, ^4S^Td and ^2S^Td are good substrates for both UP and TP; moreover, ^2S^U, ^4S^Td and 2’-deoxy-5-azacytidine (Decitabine) are substrates for PNP and the phosphorolysis of the latter is reversible. Condensation of ^2S^Ura and 5-azacytosine with dRib-1P (Ba salt) catalyzed by the accordant UP and PNP in Tris∙HCl buffer gave ^2S^Ud and 2’-deoxy-5-azacytidine in 27% and 15% yields, respectively. 6-Azauracil and 6-azathymine showed good substrate properties for both TP and UP, whereas only TP recognizes 2-thio-6-azathymine as a substrate. 5-Phenyl and 5-*tert*-butyl derivatives of 6-azauracil and its 2-thioxo derivative were tested as substrates for UP and TP, and only 5-phenyl- and 5-*tert*-butyl-6-azauracils displayed very low substrate activity. The role of structural peculiarities and electronic properties in the substrate recognition by *E. coli* nucleoside phosphorylases is discussed.

## Introduction

Nucleosides of 4- and 2-thioxopyrimidines and 6-azapyrimidines attract much attention from the time of pioneering works in the early 1950s on the chemical synthesis and investigation of their physicochemical and biological properties (early works are reviewed in [1–2]). Studies on thioxo- and azapyrimidine nucleosides are an inspiring subject of investigation due to their very special biochemical [3–7] and biophysical properties in comparison with the natural pyrimidine nucleosides in order to understand the impact of such modifications as monomers or constituents of oligonucleotides [8–14]. Thioxopyrimidine nucleosides as such, as well as building blocks of artificial oligonucleotides demonstrate promising antiviral activity in various experiments [15–22].

Regarding the chemical synthesis of this class of pyrimidine nucleosides various approaches were published (see, e.g., [8–11,14–16,23–24]; reviewed by Vorbrüggen and Ruh-Pohlenz [25]). On the contrary, only few publications are available on the enzymatic synthesis of these nucleosides. W. H. Prusoff reported on the first efficient transformation of 6-azathymine into its 2'-deoxy-D-riboside in phosphate buffer (50 mM, pH 8.0; 37 °C) in the presence of thymidine as a pentofuranose donor and washed cells or cell-free extract of *Streptococcus faecalis* as biocatalysts [26]. Later on, the conversion of 6-azapyrimidines into their ribonucleosides was observed during the cultivation of *Streptococcus faecalis* [27–28] and *E. coli* [29] cells, as well as an enzymatic glycosylation of ^14^C-labeled 6-azapyrimidines employing the preparation of trans-*N*-deoxyribosylase from *Lactobacillus helveticus* NCIB 6557 [30] had been described [31]. As for an enzymatic synthesis of thioxopyrimidine nucleosides, Kalckar [32] as well as Friedkin and co-workers [33–34] disclosed the formation of 2-thiouracil riboside and 2'-deoxyriboside using 2-thiouracil and the corresponding α-D-pentofuranose-1-phosphates (dicyclohexylammonium salts) as substrates of horse liver thymidine phosphorylases (hlTP) (reviewed in [1]).

Recently, Hatano et al. reported on the synthesis of 2-thiothymidine (^2S^Td) and 1-(2-deoxy-β-D-ribofuranosyl)-2-thiouracil (^2S^Ud) by the transglycosylation reaction of the corresponding thioxopyrimidines employing thymidine as a donor of the carbohydrate residue and *E. coli* TP as a biocatalyst [35]; 6-azauracil (**3a**) and 6-azathymine (**4a**) displayed no substrate activity in analogous reactions (cf. [26–29]). These studies prompted us to investigate the enzymatic transformations of 2(4)-thioxo- and 6(5)-azapyrimidines and their nucleosides in more detail and to outline (i) the scope and limitations of the enzymatic synthesis of 2'-deoxy-β-D-ribonucleosides catalyzed by the recombinant *E. coli* uridine (UP; EC 2.4.2.3) and thymidine (TP; EC 2.4.2.4) phosphorylases [36], and (ii) the role of structural features and electronic properties of the pyrimidine bases and nucleosides in the recognition by *E. coli* nucleoside phosphorylases.

## Results and Discussion

**Scheme 1:**
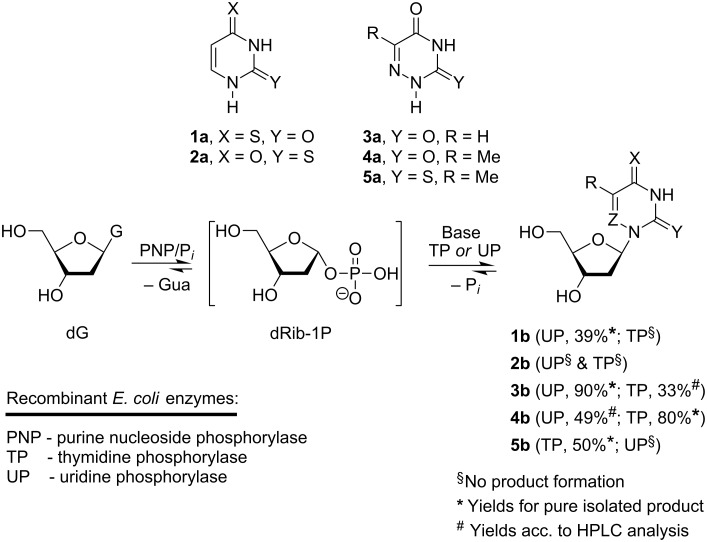
Enzymatic synthesis of 2-deoxy-β-D-ribofuranosides **1b**–**5b** of the heterocyclic bases **1a**–**5a**. Regents and conditions: dG/base ratio: 1.5:1.0 (mol), 10 mM K,Na-phosphate buffer; 40 °C, 48–72 h; HPLC analysis of the reaction mixtures see Experimental section and Supporting Information File 1.

**Thioxo- and 6-aza-pyrimidines used in the transglycosylation reaction with recombinant *E. coli* nucleoside phosphorylases and 2'-deoxyguanosine as a donor of 2-deoxy-D-ribofuranose:** The substrate properties of 4-thiouracil (**1a**; ^4S^Ura), 2-thiouracil (**2a**; ^2S^Ura), 6-azauracil (**3a**), 6-azathymine (**4a**) and 6-aza-2-thiothymine (**5a**) for the recombinant *E. coli* TP and UP in the transglycosylation reaction [37–38] using 2'-deoxyguanosine (dG) as a donor of the pentofuranose moiety in a combination with the recombinant *E. coli* purine nucleoside phosphorylase (PNP; product of *deoD* gene; EC 2.4.2.1) [36] were tested under standard reaction conditions and then individual nucleosides **1b**, **3b**–**5b** were prepared and their structure was proved by the integrity of spectral methods and comparison with the published spectral data. The most essential synthetic results are shown on Scheme 1, ^1^H and ^13^C NMR data are presented in Supporting Information File 1, Tables S1 and S2.

**Enzymatic synthesis and phosphorolysis pathways of 4(2)-thioxopyrimidine nucleosides**: 4-Thiouracil (**1a**; ^4S^Ura) revealed satisfactory substrate activity for *E. coli* UP giving rise to the formation of 4-thio-2'-deoxyuridine (**1b**; ^4S^Ud) that was prepared in 39% yield (not optimized). Unexpectedly, ^4S^Ura did show complete inertness for TP. Neither UP nor TP were able to catalyze the transformation of 2-thiouracil (**2a**) into 1-(2-deoxy-β-D-*erythro*-pentofuranosyl)-2-thiouracil (^2S^Ud; **2b**) under analogous reaction conditions (Scheme 1). The substrate properties of 4-thiouracil clearly point to essential differences in the modes of substrate binding and activation at the catalytic sites of UP and TP. Whereas the substrate recognition of 4-thiouracil by the latter enzyme depends strongly on the electronic structure and/or the van der Waals radius of the substituent at C-4, UP is less sensitive to these differences, in particular, to modification at the C-4 carbonyl group.

**Scheme 2:**
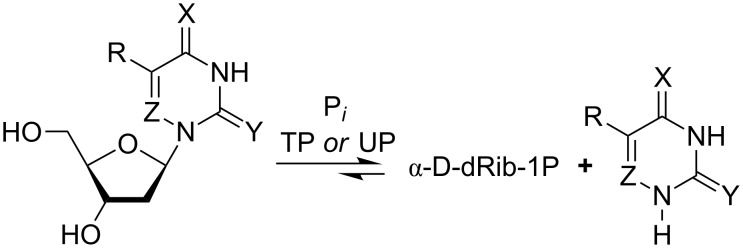
Phosphorolysis of nucleosides **1b**–**5b** and related pyrimidine nucleosides (2’-deoxyuridine, thymidine, 2- and 4-thiothymidines) catalyzed by *E. coli* UP (Figure 1) and TP (Figure 2).

**Figure 1:**
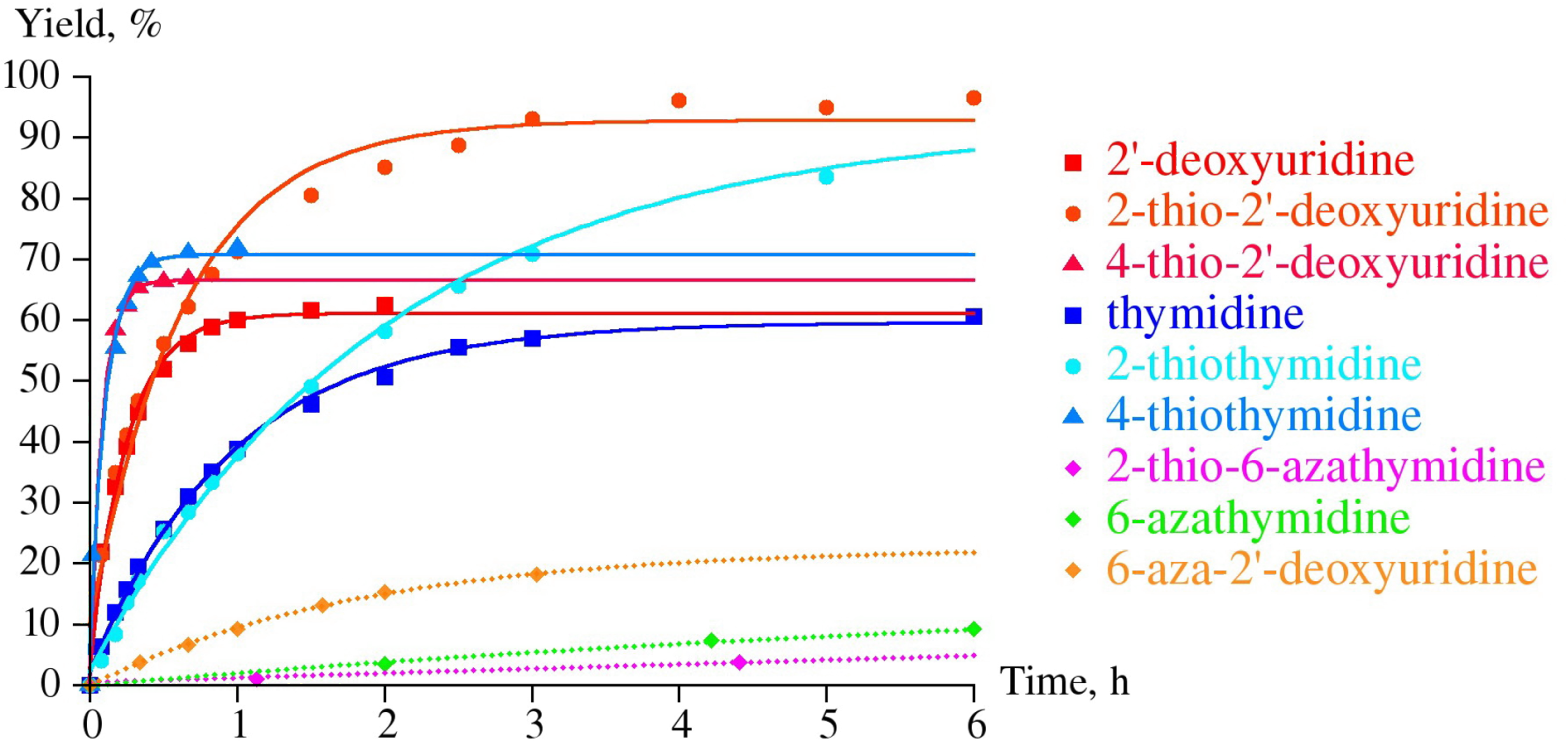
Phosphorolysis of a number of 2’-deoxy-β-D-ribofuranosides of uracil and thymine, and their 6-aza derivatives in comparison with the corresponding 4- and 2-thio derivatives catalyzed by *E. coli* UP. Reaction conditions: reaction mixture 1.0 mL; 25 mМ K,Na-phosphate buffer (рН 7.0), 20 °C; 2 mМ testing nucleoside. Enzyme: 0.016 units of *E. coli* UP (substrates drawn with solids lines) or 1.9 units of *E. coli* UP (substrates drawn with dotted lines); reaction progress was monitored by HPLC (see Supporting Information File 1); yields refer to the percentage of the resulting heterocyclic base.

**Figure 2:**
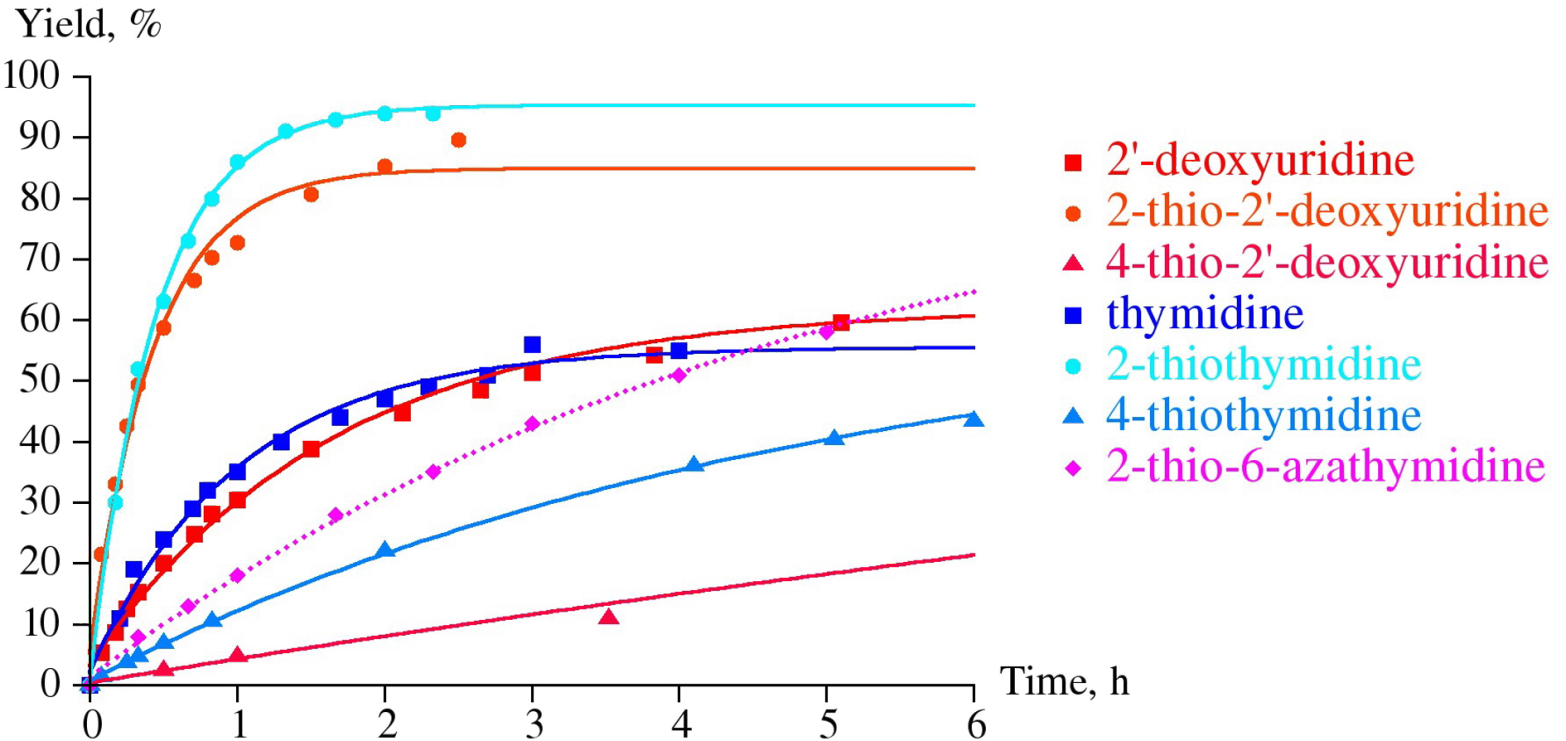
Phosphorolysis of 2′-deoxyuridine and thymidine, their 4- and 2-thio derivatives and 6-aza-2-thiothymidine (**5b**) catalyzed by *E. coli* TP (for reaction conditions, see caption of Figure 1. Enzyme*:* 6.6 × 10^−4^ units of TP was used for all substrates, except for 6-aza-2-thiothymidine, for which 26.5 units of the enzyme was used. Phosphorolysis of 6-aza-2′-deoxyuridine and 6-azathymidine did not exceed a few percent even at high enzyme concentrations and is not presented on the plot.

The results of the reverse reaction, the phosphorolysis of 4-thio-2'-deoxyuridine (**1b**; ^4S^Ud) and 4-thiothymidine (**11a**, ^4S^Td) by UP and TP, are in good agreement with the data on the synthesis (Scheme 2; Figure 1 and Figure 2). Indeed, the phosphorolysis of ^4S^Ud and ^4S^Td by UP proceeds very quickly and reaches equilibrium in the reaction ^4S^Ura (^4S^Thy) + dRib-1P 
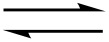
 ^4S^Ud (^4S^Td) + inorganic phosphate (P*_i_*) [base–nucleoside ratio 65(70):35(30)] within several minutes. The phosphorolysis pattern for ^4S^Ud is in satisfactory agreement with the net output (39%) of individual nucleoside obtained in the synthesis. Notably, phosphorolysis of (i) 2'-deoxyuridine (Ud) by UP proceeds at somewhat lower rate, but also reaches an equilibrium point at a similar ratio of uracil-Ud within one hour and then remains practically unchanged, and (ii) thymidine (Td) occurs at a lower rate than Ud and significantly slower than ^4S^Ud, but comes to the similar ratio of the starting substrate and thymine formed after 10 h (Figure 1).

On the contrary, (i) the close similarity of substrate activity of Ud and Td towards TP was observed, and (ii) the TP catalyzed phosphorolysis of 4-thiothymidine (**11a**, ^4S^Td) and 4-thio-2'-deoxyuridine (**1b**; ^4S^Ud) proceeds very slowly suggesting the negative impact of the 4C=O → 4C=S replacement on the binding or/and activation of both pairs of substrates 4-thiothymine (^4S^Thy) and 4-thiothymidine (^4S^Td), and, to a greater extent, 4-thiouracil (^4S^Ura) and its 2'-deoxyriboside ^4S^Ud (Figure 2).

The inertness of ^2S^Ura towards glycosylation catalyzed by both UP and TP was surprising in the light of the works of Kalckar and Friedkin which studied TP from horse liver [1,32–34]. Moreover, Hatano et al. [35] briefly described a very efficient conversion of 2-thiouracil and 2-thiothymine into the corresponding 2'-deoxyribosides in 54 and 61% yields, respectively, employing thymidine as a donor of the pentofuranose moiety and TP (Sigma) as a biocatalyst; the TP quantity and the reaction conditions are not indicated. The discrepancy between our results and those by Hatano et al. remains unclear. To shed more light into the problem, we reexamined the phosphorolysis profiles of 2'-deoxy-2-thiouridine (**2b**; ^2S^Ud) and 2-thiothymidine (**11b**; ^2S^Td) by *E. coli* UP and TP and compared them with those for corresponding natural nucleosides. We observed that the phosphorolytic cleavage of the glycoside linkages (i) of 2-thioxo analogues and corresponding natural nucleosides by UP proceeds at the same rate at the initial stages of reactions, (ii) of Ud and ^2S^Ud occurs somewhat faster vs that of Td and ^2S^Td, and (iii) of Ud and Td stops at the level of ca. 55% conversion. Contrary to the natural substrates, the reactions of ^2S^Ud and ^2S^Td continued and reached equilibrium at a ratio of substrates ca. 95:5 after 5–7 h (Figure 1). As distinct from UP, phosphorolysis of ^2S^Ud and ^2S^Td catalyzed by TP proceeded much more quickly vs the corresponding natural nucleosides attaining 85–95% conversion in ca. 2 h (Figure 2). In general, replacement of the 2-oxo group by a thioxo function leads to a significant increase of the rate of phosphorolysis of ^2S^Ud and ^2S^Td by *E. coli* UP (conversion 80–90% in 5–6 h) and to a greater extent, by TP (conversion 90–95% in 2–2.5 h) compared with the respective natural substrates.

In addition, a number of experiments on the synthesis of ^2S^Ud have been conducted and it was verified that (i) the use of thymidine as a 2-deoxy-D-ribofuranose donor (thymidine:^2S^Ura ratio of 2.5:1 (mol); rt, 24 h) at decreasing molarity of phosphate buffer to 0.4 mM (pH 7.0) resulted in the formation of ^2S^Ud in 3% and 25% yields (HPLC) in the reactions catalyzed by UP and TP, respectively; (ii) the reaction of ^2S^Ura and 2-deoxy-α-D-ribofuranose-1-phosphate and *E. coli* UP in Tris∙HCl buffer resulted in an equilibrium of starting ^2S^Ura and formed ^2S^Ud of ca. 7:3 in the reaction mixture after 1 h, from which the desired nucleoside was isolated by column chromatography in 27% yield (see Experimental section).

**The role of hydrogen bonding between Gln166 and the pyrimidine base in the interaction with** ***E. coli*** **UP**: The *E. coli* UP catalyzes the reversible phosphorolysis of a number of the base and pentofuranose modified pyrimidine nucleosides to the corresponding bases and α-D-pentofuranose-1-phosphates (α-D-PF-1P) (reviewed in [1–2,37–38]). The crystal structure of *E. coli* UP in a complex with substrates was analyzed (see [39–40] and the works cited therein) and the role of some amino acid residues of the catalytic site was characterized by the single-site mutagenesis [41]. It was suggested that the uracil binding site includes Gln166, the carboxamide group of which forms two strong hydrogen bonds 3N(H)···(O=)C(R)-NH_2_···(O=)C-2. Moreover, the carbonyl group of the Glu166 side-chain forms an additional hydrogen bond with a water molecule, which, in turn, takes a part in hydrogen bonding to the 4C(=O) oxo function of uracil and to the adjacent guanidinium group of Arg223 [40]. In addition, the side-chain of Arg168 is directly hydrogen-bonded to the 4C(=O) of uracil and the hydrogen bonds network of the side-chains of Gln166, Arg223 and Arg168 are responsible for the strict specificity to uracil and to a lesser extent to thymine [5–6] recognizing the 2C(=O)-3N(H)-4C(=O) fragment as distinct from the 2C(=O)-3N=4C(NH_2_) part of cytosine. Notably, the eight-membered ring comprising two H-bonds formed by the side-chain of Gln166 plays an important role in the phosphorolytic cleavage of the glycoside bond and in all likelihood in the reversed reaction of the glycoside bond formation. It is conceivable that the Gln166/base hydrogen bonding provides the correct positioning of uracil and its closely related analogues, e.g., 5-fluorouracil, thymine and its 5-trifluoro analogue etc, within the catalytic site of *E. coli* UP, which is accompanied by the substrate activation ensuring the subsequent attack of the N-1 nitrogen atom on the electrophilic C-1 atom of α-D-PF-1P. In the case of *E. coli* TP, there are controversial suggestions regarding the amino acid residues participating in the nucleoside binding and activation at the catalytic site precluding the similar analysis based on the most important interaction (vide infra).

**Suggested mechanistic differences of tautomeric structures of pyrimidines studied and their recognition by *E. coli* UP vs TP in the glycoside bond formation**: Activation of uracil and thymine as well as their related analogues in the chemical synthesis of nucleosides consists in the trimethylsilylation giving rise to the formation of 2,4-di-O-TMS derivatives accompanied by the sp^3^ → sp^2^ transformation of the nitrogen atoms. The sp^2^ hybridized nitrogen atoms attack the electrophilic C-1 carbon atom of the appropriately protected sugar derivatives leading to the glycosylic bond formation (reviewed in [25]). A similar mechanism occurs during the attack of the sp^2^ hybridized N-1 atom of the pyrimidine base to the anomeric C-1 carbon atom of α-D-PF-1P in the nucleophilic substitution of phosphoric acid residue of the latter catalyzed by *E. coli* UP and TP.

It was unequivocally proven by the physicochemical and theoretical methods that natural pyrimidine bases exist in the gas phase and in water solution in the canonical 2,4-diketo form (e.g. [42–43]). The tautomeric structures of 4- and 2-thioanalogues of thymine and uracil have been also investigated by diverse methods and the predominant population of the oxo/thioxo tautomers was established [44–47]. We have analyzed the electronic structures of 2,4-diketo and regioisomeric oxo/thioxo tautomers of uracil, 4-thiouracil and 2-thiouracil, as well as their 4(2)-enol(mercapto) forms by the ab initio method (6-31G** level; basic set of parameters). We found that oxo/thioxo structures are thermodynamically more stable by 10–15 kcal/mol on all the occasions; similar analysis of 6-azapyrimidines showed even higher thermodynamic stability of the keto/thioketo tautomers by 20–30 kcal/mol (Supporting Information File 1, Table S3).

**Figure 3:**
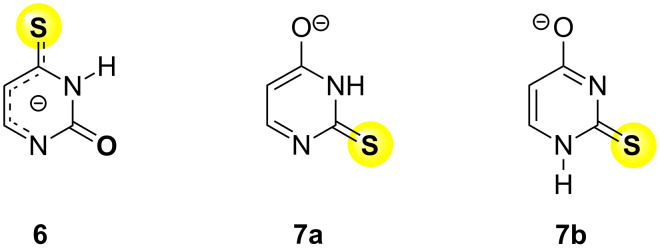
Supposed monoanionic forms of 4-thiouracil and 2-thiouracil in aqueous medium [48–49].

Furthermore, the monoanionic forms of 4-thiouracil and 2-thiouracil in aqueous medium were analyzed by the UV and IR spectroscopic methods and the structure **6** with charge delocalization for the former and two tautomeric monoanions **7a** and **7b** (ca. 1:1) with charge localization on the C-4 oxygen atom for the latter were suggested [48–49] (Figure 3).

The aforementioned observations, as well as the very likely main contribution of the side-chain of Glu166 in the correct positioning of a pyrimidine substrate at the *E. coli* UP catalytic site suggests a possible mechanism of the activation of the substrate and we became interested in whether this interaction contributes to the sp^3^ → sp^2^ transformation of the N-1 nitrogen atoms. With this aim in view, we constructed and geometry optimized [Bio+(CHARMM27) force field] and then re-optimized using semi-empirical PM3 method (HyperChem 8.1) the eight-member cyclic structures comprising two hydrogen bonds between acetamide (AA) as a Gln166 side-chain mimic and tautomers of bases with sp^3^ (**8a**) vs sp^2^ (**8b** and **8c**) hybridized N-1 nitrogen atoms (Table 1; for details, see Supporting Information File 1, Table S4).

**Table 1:** Geometry optimized supposed tautomeric structures of uracil and bases **1a**–**5a** at *E. coli* UP catalytic site with sp^3^ and sp^2^ hybridized N-1 nitrogen atoms (thermodynamically stable tautomers are highlighted in boldface, and the more stable N-1 sp^2^ structures are in italics and underlined)^a^.

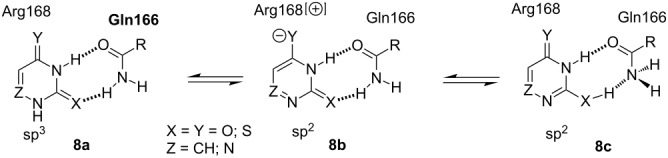							

Substrate	*E*_TOTAL_ kcal/mol (Δ*E*_TOTAL_)^b^	Partial charge of the N-1 atom (*e*)	*E*_TOTAL_ kcal/mol (Δ*E*_TOTAL_)^b^	Partial charge of the N-1 atom (*e*)	*E*_TOTAL_ kcal/mol (Δ*E*_TOTAL_)^b^	Partial charge of the N-1 atom (*e*)	Remarks

uracil	**–51,160.1**	**0.083**	*−51,151.2* (+8.9)	*−0.202*	−51,144.0 (+16.1)	−0.214	UP and TP accept uracil as a substrate
4-thiouracil (**1a**)	–48,654.5 (+6.5)	0.114	**−48,661.0**	**−0.166**	**−**48,640.4 (+20.6)	**−**0.167	only UP recognizes **1a** as a substrate
2-thiouracil (**2a**)	**–48,657.0** (+0.7)	**0.197**	**−**48,644.0 (+13.0)	**−**0.128	**−48,657.7**	**−0.146**	TP recognizes **2a** better vs UP
6-azauracil (**3a**)	**–51,792.7**	**0.050**	**−***51,783.7 (+10.0)*	**−***0.136*	**−**51,779.9 (+12.8)	**−**0.191	UP recognizes **3a** better vs TP
6-azathymine (**4a**)	**–55,244,3**	**0.057**	**−***55,235.6 (+8.7)*	**−***0.132*	**−**55,234.0 (+10.3)	**−**0.193	TP recognizes **4a** better vs UP
6-aza-2-thio- thymine (**5a**)	**–52,739.7** (+1.1)	**0.174**	**−**52,731.1 (+9.7)	**−**0.064	**−52,740.8**	**−0.109**	only TP accapts **5a** as a substrate

^a^Only total energy values (*E*_TOTAL_, kcal/mol) and partial charges (*e*) of the N-1 atoms are given (for detailed information, see Supporting Information File 1, Table S4); thermodynamically stable tautomers are highlighted in boldface, and the more stable N-1 sp^2^ structures are highlighted in italic and underlined. ^b^The Δ*E*_TOTAL_ values (are given in parenthesis; kcal/mol) mean the differences between the *E*_TOTAL_ values of the thermodynamically most stable tautomer and the less stable tautomers.

The uracil/AA tautomer with the sp^3^ hybridized N-1 atom is thermodynamically more stable by 9 and 16 kcal/mol vs those of the N-1 sp^2^ structures implying that (i) the base/Gln166 hydrogen bonding alone is not apparently sufficient to realize the sp^3^ → sp^2^ transformation, and (ii) the concerted interaction of side-chains of the triad Gln166, Arg223 and Arg168 of the UP catalytic site makes it possible to overcome the barrier of 9 kcal/mol required to activate the substrate. At the same time, the corresponding sp^2^ structures **8b** and **8c** are characterized by the highest partial charges of the N-1 atoms (−0.202/−0.214 e) reflecting the nucleophilicity of these atoms that attack the electrophilic C-1 of α-D-PF-1P.

**Figure 4:**
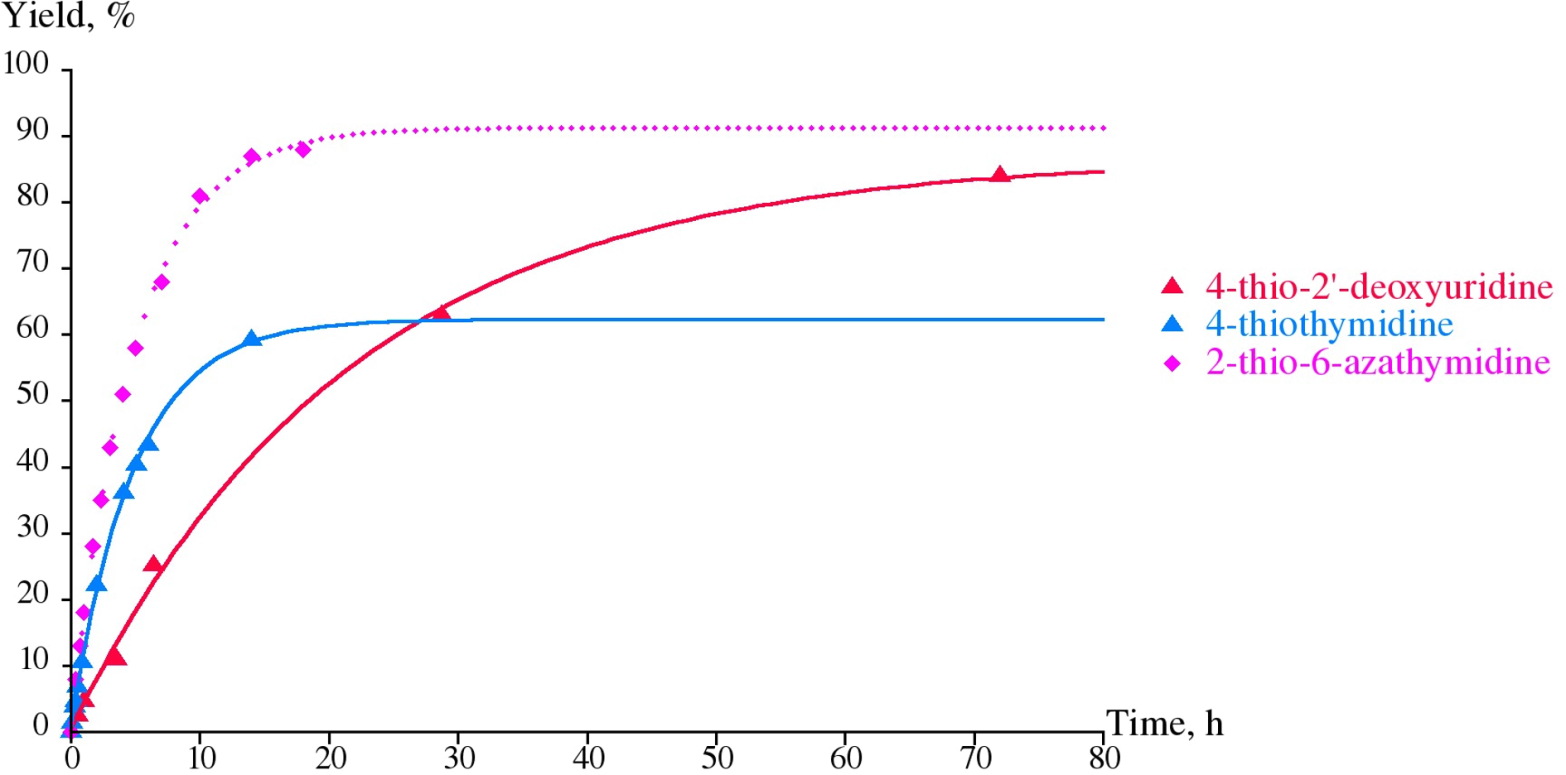
Phosphorolysis of 6-aza-2-thiothymidine (**5b**), 4-thiothymidine (**11a**) and 4-thio-2′-deoxyuridine (**1b**) by *E. coli* TP for extended time period (for details, see caption of Figure 2).

Among the bases analyzed, only the sp^2^ N-1 form of the 4-thiouracil/AA structure **8b** is thermodynamically favorable over the N-1 sp^3^ tautomer **8a** by −6 kcal/mol and the partial charge of the N-1 is calculated to be −0.166 e, which is compatible with its satisfactory substrate activity for *E. coli* UP. Under similar reaction conditions, 4-thiouracil revealed no substrate activity against *E. coli* TP suggesting the various types of the substrate binding and activation at *E. coli* UP and TP catalytic sites in spite of the some similarity of the interaction of the respective side-chains of Arg168 (UP) [40] and Arg171 (TP) [50] with a substituent at the C-4 atom. Notably, phosphorolysis of ^4S^Td and ^4S^Ud by TP (Figure 2 and Figure 4) proceeds essentially slower vs the one catalyzed by UP (Figure 1). It appears to be reasonable that TP manifests strict requirements to dimension and electronic structure of the C-4 substituent [4], as well as the TP catalyzed transformations depend in a greater extent on the experimental condition [51].

The formation of the productive complex matching the structure **8b** at the catalytic site of *E. coli* UP [40] corresponds well to the spectroscopic data for the mono-anionic form of the tautomer **6** of 4-thiouracil [48–49] (Figure 3). Decrease in the rate of the transglycosylation reaction of 4-thiouracil vs uracil can be explained by the decrease of the hydrogen binding capacity of C-4 sulfur atom compared with the oxygen (cf. the corresponding data for uracil and 4-thiouracil, Supporting Information File 1, Tables S3 and S4) and to some extent by a distortion of architecture of the productive complex due to differences of the van der Waals radii of the substituents at the C-4 atom.

In the case of 2-thiouracil, two tautomeric structures **8a** and **8c** are characterized by rather similar thermodynamic parameters, which implies the possibility of the binding and activation of 2-thiouracil due only to the base/Gln166 hydrogen bonding at the *E. coli* UP catalytic site. Unfortunately, there exist different assumptions concerning the amino acid residues of the *E. coli* TP catalytic site that are directly involved in the hydrogen bonding with pyrimidines and types of these interactions [3,52–56] (vide infra). Indeed, except for the side-chain Arg171/C-4 carbonyl interaction, several variants of direct hydrogen binding between the side-chains of Ser186 and Lys190 and the respective 3N(H) and 2C(=O) functions of substrate (open form) and its activation (catalytically competent closed form) are proposed, wherein there is no prevailing factor determining the substrate recognition (as opposed to *E. coli* UP), giving an impetus to the sp^3^ → sp^2^ transformation of the N-1 nitrogen atom. Moreover, Gago et al. had analyzed the possibility of changing the direct 2C(=O)/Lys190 (side-chain) interaction of thymidine in the open form on the mixed type of the direct hydrogen bonding of C-2 carbonyl with the side-chains of Lys190 and His85 in a transitional state and then to the direct 2C(=O)/His85 hydrogen bonding of thymine (product of reaction) in a closed form [3] (cf. [52,55]). This uncertainty does not allow identifying the most important type of hydrogen bond and analyzing its impact on the N-1 sp^3^ → sp^2^ transformation of the substrate.

**The enzymatic synthesis of 6-azapyrimidine nucleosides: the C-2 thioxo motif in the reactions catalyzed by *E. coli* nucleoside phosphorylases**: Like uracil, complexes of 6-azauracil (**3a**; aUra) and 6-azathymine (**4a**; aThy) with Gln166 with sp^3^-hybridized bases of the type **8a** are thermodynamically more stable (Table 1); each of the bases is a substrate for both enzymes, UP and TP, and the glycosylation efficiency depends, as might be expected, on the C-5 substituent of the heterocycle, viz., glycosylation of 6-azauracil occurs more efficiently under UP catalysis, whereas the 6-azathymine synthesis proceeds more efficiently in the presence of TP and these reactions strongly displaced to the nucleoside formation (Scheme 1).

As distinct from 2-thiouracil, 6-aza-2-thiothymine (**5a**) was found to be a satisfactory substrate for *E. coli* TP and 6-aza-1-(2-deoxy-β-D-*erythro*-pentofuranosyl)-2-thiothymine (**5b**) was prepared in 50% yield (not optimized). Analysis of 6-aza-2-thiothymine/Gln166 complexes revealed that, like 2-thiouracil, more stable is the structure **8c**; however, unlike 2-thiouracil and 6-azathymine, only TP catalyzed reversible glycosylation of **5a** (see phosphorolysis of **5b**, Figure 2 and Figure 4) that proceeds satisfactorily. On the contrary, UP is not able to catalyze the nucleophilic attack of the structure **8c** of ^2S^aThy owing probably to the lower nucleophilicity of the N-1 nitrogen atom (−0.109 e) compared to ^2S^Ura for which the partial charge of −0.146 e of the N-1 atom was calculated (Table 1). With respect to *E. coli* TP, the glycosylation of 6-azathymine (**4a**) appears to proceed virtually irreversibly (data not shown) with somewhat higher efficacy compared to 6-aza-2-thiothymine (**5a**) and, as a consequence, results in the high yield of the desired 2'-deoxyriboside **4b**. Replacement of the C-2 oxygen atom of 6-azathymine (**4a**) by a sulfur atom changes the pattern of the TP catalyzed glycosylation of 6-aza-2-thiothymine (**5a**), imparting the reaction reversible character (Figure 4).

UP was practically unable to catalyze the synthesis of the nucleoside **5b** (Figure 1). Again, unlike the base/Gln166 H-bonding mode, another kind of the interaction of the C-2 thioketo or mercapto function with the side-chain(s) of amino acid residue(s) of the TP active center plays a crucial role in the establishment of the observed **5a** + dRib-1P 
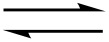
 **5b** + P*_i_* equilibrium.

As mentioned above, we did not observe the formation of 1-(2-deoxy-β-D-*erythro*-pentofuranosyl)-2-thiouracil (^2S^Ud; **2b**) under standard reaction conditions of the *trans*-2'-deoxyribosylation (Scheme 1), contrary to the previously published data [1,33–35] and speculated that in our experiment, the reaction equilibrium is almost completely displaced to the starting substrates, ^2S^Ura and dRib-1P (vide supra). Indeed, we have earlier proved that replacement of the C-2 carbonyl group of 1-(2,3-dideoxy-3-fluoro-β-D-*erythro*-pentofuranosyl)thymine (FLT), which is strongly resistant towards *E. coli* TP [16,57], with the thiocarbonyl group gives 2-thio-FLT derivative that is phosphorolyzed by *E. coli* TP [16]. Later on, Gago et al. found that *E. coli* TP showed lower affinity (*K*_m_) for 2-thiothymidine vs that of thymidine (1.587 vs 0.8 mM), and vice versa maximal initial velocity value (*V*_max_) for ^2S^Td is higher (65.4 vs 24.2 nanoM × mL^−1^·min^−1^) pointing to a higher substrate activity of ^2S^Td vs that of thymidine in the phosphorolysis. In the present paper, we have shown that in the reactions catalyzed by UP, conversion of natural substrates, Ud and Td, to products stops at ca. 55%, while the phosphorolysis of the corresponding C-2 thio analogues reaches more than 90% (Figures 1, 2 and 4). In this regard, the absence of substrate activity of 2-thiouridine towards UP and TP observed recently [5,58] appears to be completely unexpected since it implies a dramatic change of the substrate specificity of the enzyme.

**Figure 5:**
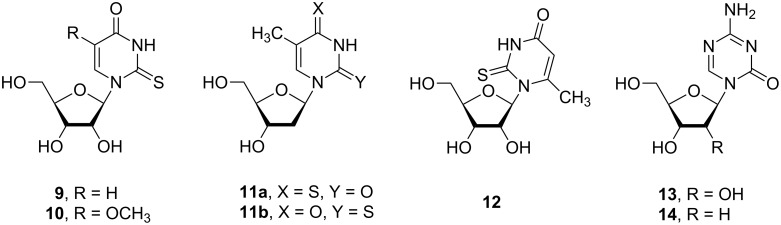
Structures of 2-thiopyrimidine(**9**–**12**) and 5-azacytidine (**13** and **14**) nucleosides.

**Substrate properties of related nucleosides and bases for *E. coli* UP, TP and PNP**: The aforementioned contradictory data prompted us to test the substrate properties of 2-thiouridine (**9**), 2-thio-5-methoxyuridine (**10**), 4-thiothymidine (**11a**), 2-thiothymidine (**11b**), 6-methyl-2-thiouridine (**12**), 5-azacytidine (**13**; aC) and 5-aza-2′-deoxycytidine (**14**; aCd; anticancer drug Decitabine) (Figure 5) [25,59] for *E. coli* UP, TP and PNP (for reaction conditions, see Experimental section).

**Figure 6:**
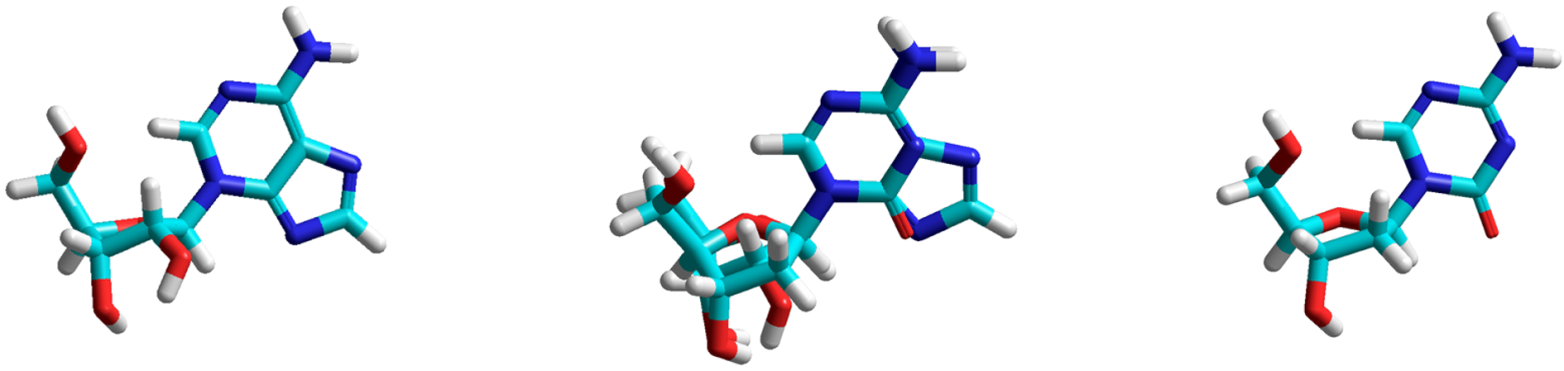
Energy minimized structures of *N*^3^-(β-D-ribofuranosyl)adenine (left) and 5-aza-2′-deoxycytidine (right) and in the mid both structures are overlapped by the glycosyl bonds (calculations by ab initio, 3-21G level; Polak–Ribiere (conjugate gradient); basis set of parameters; HyperChem 8.1).

We found that compounds **9**–**11a,b** are good substrates for both UP and TP (see also Figures 1, 2 and 4); 6-methyl-2-thiouridine (**12**) showed no substrate activity for both nucleoside phosphorylases. This latter observation points to the importance of the *syn*/*anti* base orientation around the glycosyl bond in the definition of the substrate properties of pyrimidine nucleosides towards UP and TP. It should be noted that (i) 6-methyl-2'-deoxyuridine (^6Me^Ud) was shown to undergo an irreversible phosphorolysis in the *E. coli* living cells or in the presence of non-dialyzed cell-free extract from *E. coli* B and the 15^T^-mutant [60] and (ii) 6-methyluridine is very week substrate for UP [42,61]. Analysis of the NMR spectra of ^6Me^Ud in D_2_O pointed to the predominant base population in the *syn*-conformation [62]. The stereochemistry of 6-methyluridine was also investigated by physicochemical methods [63–65] and in crystal [66] and the dominating *syn* base conformation around the glycosyl bond was established.

We have earlier shown that 2'-deoxy-β-D-ribosides of cytosine, uracil and thymine are substrates for *E. coli* PNP (Cd >>> Ud >> Td), whereas the corresponding ribosides devoid of substrate activity [67]. The substrate activity of 5-aza-2'-deoxycytidine (**14**) (but not 5-azadeoxycytidine (**13**)) and 4-thiothymidine (**11a**) for PNP (aCd >>> ^4S^Td) correlate well with that of the corresponding natural 2-deoxyribosides. Unexpectedly, 2-thiouridine (**9**) showed weak activity for PNP whereas 2-thiothymidine (**11b**) entirely was lacking such an activity. It is conceivable that in the case of such unusual substrates of PNP as pyrimidine nucleosides their structural features define the substrate properties, viz., 2-thiouridine (very weak substrate) vs uridine (non-substrate), thymidine (very weak substrate) vs 2-thiothymidine (non-substrate).

Shugar and co-workers studied the substrate properties of N-3-regioisomers of adenosine and inosine, *N*^3^-(β-D-ribofuranosyl)-adenine (*N*^3^-Ado) and -hypoxanthine (*N*^3^-Ino), respectively, towards the calf-spleen and *E. coli* PNPs and disclosed their satisfactory substrate activity [68]. The authors discussed the results in terms of similar spatial organization of natural nucleosides and their N-3-isomers admitting the binding and activation of the latter at the catalytic sites of the PNPs. Noteworthy that phosphorolysis of both N-3-regioisomers proceeds irreversibly. In the present work, we compared the energy minimized structures of *N*^3^-Ado and 5-aza-2'-deoxycytidine (**14**) and found a rather similar stereochemistry of both structures, in particular, a quite similar orientation of pyrimidine and pentofuranose rings (Figure 6). Moreover, the phosphorolysis of the latter was reversible and the reaction of 5-azacytosine with 2-deoxy-α-D-ribofuranose-1-phosphate (Ba salt) (1:1 molar ratio; 2 mM) in the presence of *E. coli* PNP (10 units) in 50 mM Tris∙HCl buffer (pH 7.0) at 20 °C for 30 min resulted in the formation of nucleoside **14** in 15% yield (HPLC; System A, *t*_R_, min: 5-azacytosine – 1.6, 5-aza-2'-deoxycytidine – 5.3).

An analogous comparative analysis of stereochemistry of *N*^3^-Ino and 2-thiouridine (**9**) gave very similar results (data not shown). It is obvious that in the case of such non-conventional substrates of *E. coli* PNP, introduction of any additional factors giving rise to spatial distortion (e.g. 2-thioxo function) will lead to the loss of substrate activity.

**Figure 7:**
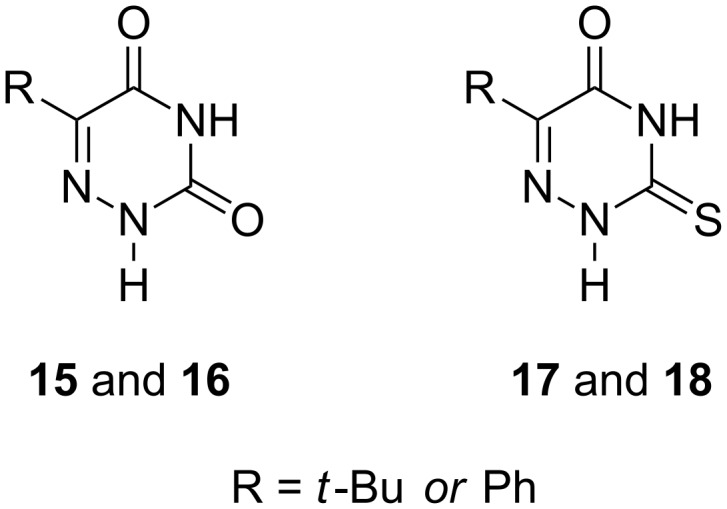
Structures of 6-azapyrimidines **15**–**18** tested for *E. coli* UP and TP.

**Substrate properties of C-5-phenyl and C-5-t-butyl substituted 6-azauracil and its C-2-thioxo derivatives**: In addition, we evaluated the tolerance of *E. coli* UP and TP with respect to the bulkiness of the substituents at the C-5 carbon atom of pyrimidines and for this purpose the substrate properties of a number of derivatives of 6-azapyrimidines **15**–**18** were tested (Figure 7).

Beginning with the pioneering works of Friedkin and co-workers (testing of 5-amino- and halogeno derivatives of uracil for TP from horse liver) and Heidelberger and co-workers (practical synthesis of 5-fluoro- and 5-trifluoromethyl-2'-deoxyurines) substrate properties of 5-substituted uracil derivatives were studied in a number of publications (reviewed in [1–2,37]) due to the great potential of these bases and nucleosides for the treatment of tumours and viral diseases. 5-Ethyl- and (*E*)-5-(2-bromovinyl)uracil and C-5 halogenated derivatives of uracil revealed good substrate activity for TP and/or UP employed within the whole *E. coli* cells and the β-D-2'-deoxyribosides of aforementioned bases have been synthesized in 55–65% yields [1,37,69]. Recently, the comparative effectiveness of immobilized pyrimidine nucleoside phosphorylase from *Bacillus subtilis* (BsPyNP) and *E. coli* TP in (i) the phosphorolysis of thymidine, uridine and its deoxy- (2'- and 5'-monodeoxy and 2',3'-dideoxy) and *arabino* derivatives, and (ii) the conversion of 5-fluoro (Br, I, CF_3_ and –CH=CHBr)-uracil into respective 2'-deoxyribosides (2'-deoxyuridine as a donor of the sugar moiety) was studied in detail by Ubiali et al. [70].

**Figure 8:**
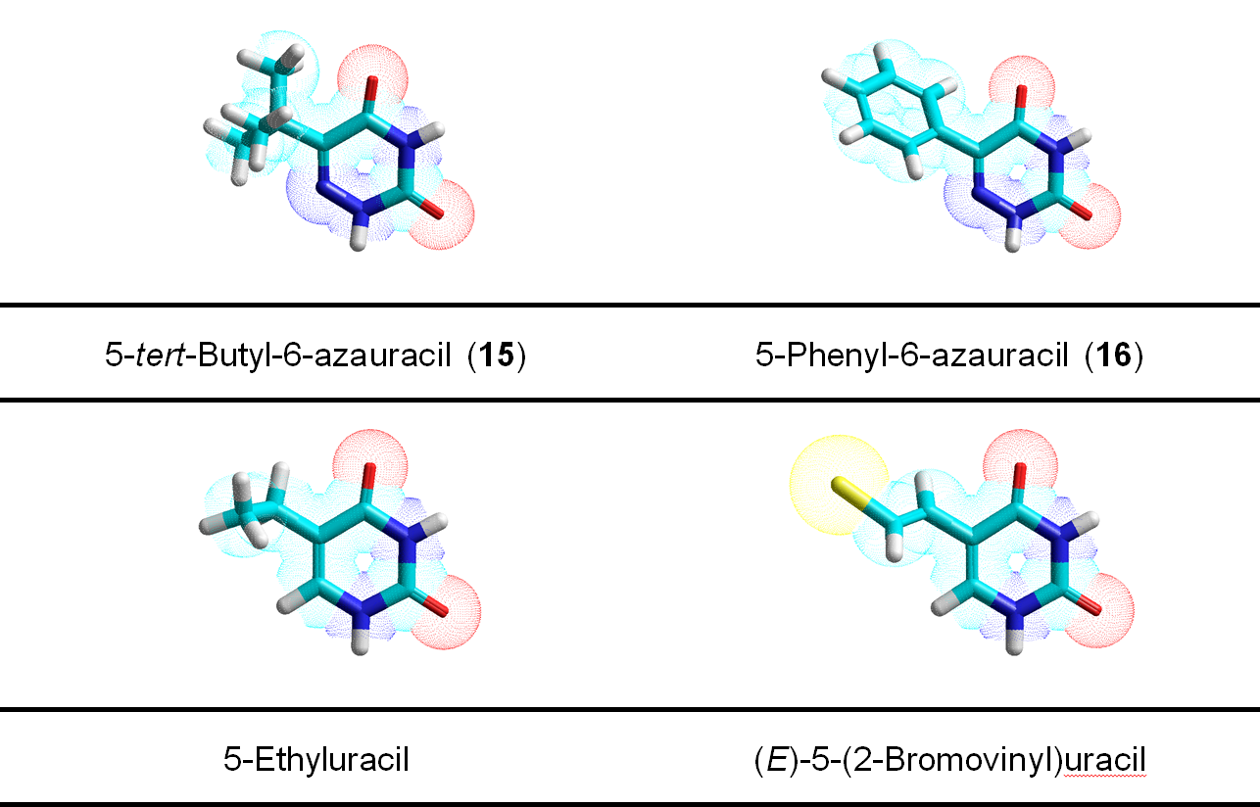
Geometry optimized structures (PM3 method) of 5-*tert*-butyl-6-azauracil (**15**) and 5-phenyl-6-azauracil (**16**) (upper structures) vs those of 5-ethyluracil and (*E*)-5-(2-bromovinyl)uracil (lower structures).

Tested in this paper 6-azapyrimidines **15**–**18** turned out to be extremely poor substrates for both *E. coli* TP and UP and the formation only 2'-deoxyribosides of 6-aza-5-*tert*-butyluracil **15** (ca. 1%) and 6-aza-5-phenyluracil **16** (ca. 2%) was detected by the HPLC/MS analysis of the respective reaction mixtures (data not shown). Thus, both enzymes demonstrated severe restrictions on the bulkiness and three-dimensional structure of the substituent at the C-5 atom of the pyrimidines. The spatial organization of the very poor substrates 5-*tert*-butyluracil (**15**) and 5-phenyluracil (**16**) vs good substrates 5-ethyluracil and (*E*)-5-(2-bromovinyl)uracil was evaluated by a geometry optimization employing the ab initio method and data are presented in Figure 8. These data suggest that the structure of the catalytically competent substrate-enzyme complex cannot accommodate spatially dispersed bases **15** and **16** in a rather tight place of the catalytic site of the enzymes.

## Conclusion

The substrate properties of 4(2)-thioxo- and 6-azapyrimidines as well as their nucleosides for *E. coli* UP and TP have been investigated leading to a number of observations that are of importance for the biotechnology of pyrimidine nucleosides. Only *E. coli* UP can effectively catalyze the *N*^1^-2'-deoxy-D-ribosylation of 4-thiouracil; 2'-deoxy-4-thiouridine (^4S^Ud) and 4-thiothymidine (^4S^Td) are excellent substrates for the enzyme, whereas phosphorolysis of Ud and Td proceeded with a somewhat lesser efficiency. ^4S^Ud and ^4S^Td revealed moderate substrate activity for *E. coli* TP. Thus, in the case of TP, the 4C(=O) → 4C(=S) replacement resulted in the loss of substrate activity of the pyrimidine base and in an decrease of substrate activity of ^4S^Td and to a greater extent of ^4S^Ud (the absence of the 5C-methyl group!) vs the relevant natural nucleosides, Td and Ud. Completely opposite effects were observed in the case of UP, viz., the substrate activity of ^4S^Ura vs that of uracil is marginally diminished (data not shown), whereas the substrate activity of ^4S^Ud and ^4S^Td is essentially enhanced (without discriminating 5C-H/Me) compared with the respective parent Ud and Td, where UP showed slight substrate preference for the former.

Replacement of the 2C(=O) carbonyl of Ud and Td with thiocarbonyl function resulted in a dramatic enhancement of the substrate activities of ^2S^Ud and ^2S^Td vs those of the parent nucleosides [5,58]. The reactions ^2S^Ura (^2S^Thy) + dRib-1P 
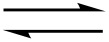
 ^2S^Ud (^2S^Td) + P*_i_* catalyzed by UP and to a greater extent by TP are displaced to the initial bases; as a consequence, ^2S^Ud was synthesized in 27% yield by the reaction of ^2S^Ura and dRib-1P in Tris∙HCl buffer in the presence of UP.

An enzymatic trans-2-deoxyribosylation of 6-azauracil (**3a**) and 6-azathymine (**4a**) catalyzed by UP and TP proceeds practically irreversibly [26–29,35]. In contrast, only TP accepts 6-aza-2-thiothymine (**5a**) as a substrate yielding its 2'-deoxyriboside **5b** as a result of the reversible glycosylation pointing again to the surprising ability of the 2C(=S) sulfur atom to enhance the substrate activity of the relevant nucleosides.

2-Thiouridine (**9**) (but not uridine [67] and 2-thiothymidine (**11b**)) and 4-thiothymidine (**11a**) (like thymidine [67]) are weak substrates for *E. coli* PNP. The most unexpected finding consists in that the phosphorolysis of 5-aza-2'-deoxycytidine (**14**; anticancer drug Decitabine) catalyzed by *E. coli* PNP is reversible and condensation of 5-azacytosine with 2-deoxy-α-D-ribofuranose-1-phosphate resulted in the formation of nucleoside **14**.

The C-5 *tert*-butyl and phenyl derivatives of 6-azapyrimidines **15** and **16** as well as their C-2 thio-counterparts **17** and **18** showed negligible substrate activity for *E. coli* UP and TP pointing to an excess of the allowed bulkiness of C-5 substituent compared with ethyl and bromovinyl groups, respectively.

## Experimental

### General methods

All chemicals and solvents were of laboratory grade as obtained from commercial suppliers and were used without further purification. The NMR and UV–vis spectra were recorded on Brucker Avance 500-DRX (Bruker, Germany) and Carry 100 spectrometers (Varian, USA), respectively. TLC was performed on TLC aluminium sheets covered with silica gel 60 F_254_. Low resolution mass spectra were measured on a LCQ Fleet ion trap mass spectrometer (Thermo Electron, USA) in 80% aq acetonitrile.

HPLC system: HPLC COMPACT Pump 2050 with Lambda 1010 UV detector (BISCHOFF Chromatography, Germany); for chromatographic conditions and retention times (*t*_R_) see Supporting Information File 1. The ^1^H and ^13^C NMR spectra of nucleosides synthesized are given in Supporting Information File 1.

Flash column chromatography was carried out on silica gel 60, 35–70 µm (Merck, USA).

The following recombinant *E. coli* enzymes [16] were used in the present study: UP, a solution in 5 mM potassium phosphate buffer (pH 7.5) with activity of 18.7 IU/mL, and TP, a solution in 5 mM potassium phosphate buffer (pH 7.0) with activity of 265 IU/mL; PNP (the product of the *deoD* gene; EC 2.4.2.1) specific activity 54 IU per mg, 17 mg per mL. All the reactions were conducted at room temperature and pH 7.0 if it is not stated otherwise.

2-Deoxy-α-D-ribofuranose-1-phosphate (barium salt) was synthesized as described previously [71].

**Initial test for substrate activity in the transglycosylation reaction:** Total volume of reaction mixture was 4 mL: 5 mM K,Na-phosphate buffer, 1 mM 2'-deoxyguanosine, 0.8 mM tested base, 40 units of PNP, 3 units of UP or TP; 40 °C. Reaction progress was monitored by HPLC (System A; see Supporting Information File 1).

**Phosphorolysis conditions** (Figures 1,2 and 4): Total volume of reaction mixture was 1 mL: 25 mM K,Na-phosphate buffer, 2 mM tested nucleoside: 0.016 units of UP or 6.6 × 10^−4^ units of TP (substrates drawn with solid lines); 1.9 units of UP or 26.5 units of TP (substrate drawn with dotted lines); room temperature. Reaction progress was monitored by HPLC (System B or C; see Supporting Information File 1).

**Substrate properties of pyrimidine nucleosides 9–14 for *E. coli* UP, TP and PNP**: Total volume of reaction mixture was 2 mL; nucleoside 1 mg/mL, 50 mM K,Na-phosphate buffer (pH 7.0), 150 IU of the corresponding enzyme, room temperature, 1 h; phosphorolysis (HPLC) of: **9** – 62% (UP), 97% (TP), 13% (PNP); **10** – 92% (UP), 96% (TP); **11a** – 34% (UP), 87% (TP), 22% (PNP); **14** – 58% (PNP); no phosphorolysis was observed in the case of UP and TP.

**4-Thio-2'-deoxyuridine (1b)**: 4-Thiouracil (100 mg, 0.780 mmol) and 2′-deoxyguanosine (313 mg, 1.17 mmol) were suspended in 10 mM K,Na-phosphate buffer (40 mL), PNP (1200 units) and UP (238 units) were added and reaction mixture was stirred at 40 °C for 48 h, and the formation of the nucleoside **1b** was monitored by HPLC (system A). After 20 h precipitated guanine was filtered off; silica gel (2 mL) was added to the filtrate and the solvent was removed in vacuo. The residue was co-evaporated with ethanol (10 mL) and put on the top of silica gel column (2 × 10 cm) that was eluted with chloroform/methanol, 30:1 (v/v). 4-Thio-2'-deoxyuridine (**1b**) was obtained as yellow powder (74 mg, 0.303 mmol, 39%), that was recrystallized from methanol; mp 164–165 °C; Lit. data [72]: 154–155 °C (from ethanol); UV (H_2_O) λ_max_, nm (ε, M^−1^∙cm^−1^): 331 (27,700) and 245 (4,800) at pH 7.0; 316 (24,600) and ca. 230 (shoulder; ca. 7,500) at pH 10.0; 331 (26,800) and 245 (4,640) at pH 4.0; λ_min_: 276 (1,600) and 225 (3,200) at pH 7.0; 258 (3,400) at pH 10.0, 276 (1,600) and 225 (3,000) at pH 4.0 [72–73]; ESIMS (positive ion mode): 267 [M + Na]^+^; ESIMS (negative ion mode): 243 [M – H]^−^

**Figure 9:**
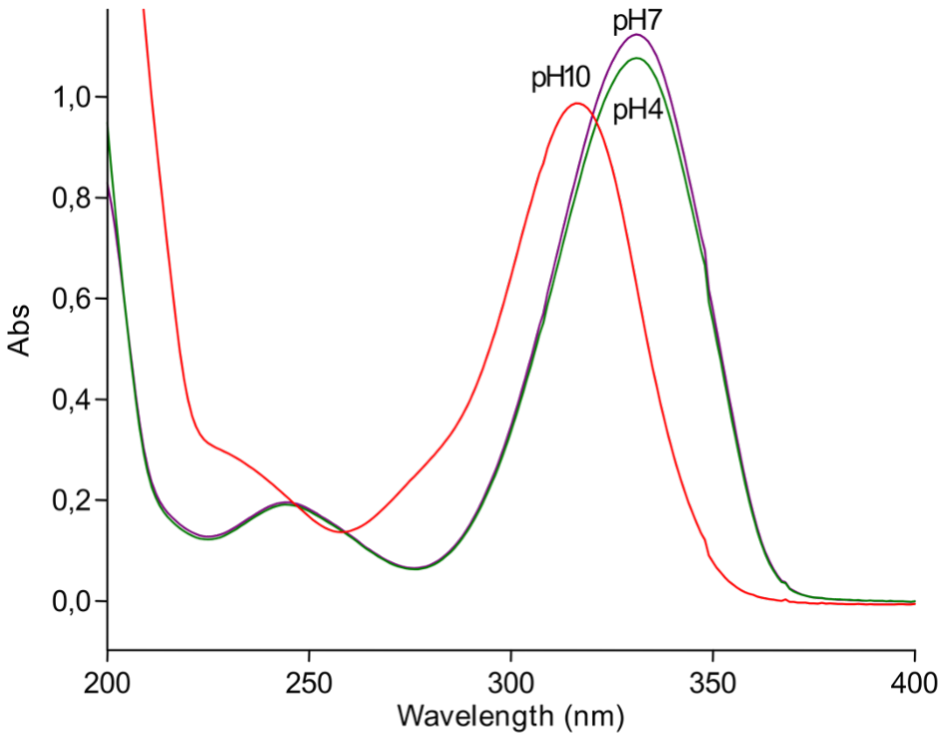
The UV spectra of 4-thio-2′-deoxyuridine (**1b**).

**2-Thio-2'-deoxyuridine (2b):** 2-Thiouracil (40 mg, 0.31 mmol), 2-deoxy-α-D-ribofuranosyl phosphate barium salt (105 mg, 0.30 mmol) and barium acetate (40 mg, 0.16 mmol) were dissolved in 20 mM Tris∙HCl buffer (pH 7.3). UP (10 units) was added, reaction mixture was gently stirred at room temperature and the formation of the nucleoside **2b** was monitored by HPLC (System C). After 1 h, equilibrium between the starting base and its nucleoside was established, the reaction mixture was filtrated, silica gel (2 mL) was added to the filtrate, and the solvent was removed in vacuo. The residue was co-evaporated with ethanol (10 mL) and put on the top of silica gel column (2 × 10 cm) that was eluted with chloroform/methanol, (10:1, v/v). 2-Thio-2'-deoxyuridine (**2b**) was obtained as white powder (20 mg, 0.097 mmol, 27%) of 98.7% purity; lit. data [74]: 133–134 °C (from abs. MeOH and technical hexane); UV (H_2_O) λ_max_, nm (ε, M^−1^∙cm^−1^): 217 (15,000) and 274 (12,950); (1 N NaOH) 238 (19,600) and 269 (19,600); UV (H_2_O) λ_max_, nm (ε, M^−1^∙cm^−1^): 272 (13,100) and 218 (14,700) at pH 7.0; 263 (13,700) and ≈240 (sh, ≈10,000) at pH 10; 275 (13,700) and 217 (16,400) at pH 4; λ_min_: 240 (8,100) at pH 7.0; 216 (9,200) at pH 10.0; 240 (8,300) at pH 4.0.; ESIMS (positive ion mode): 245 [M + H]^+^; ESIMS (negative ion mode): 195 [M − •C5'H_2_OH − •O3'H – H]^−^.

**6-Aza-2'-deoxyuridine (3b):** 6-Azauracil (106 mg, 0.937 mmol) and 2'-deoxyguanosine (376 mg, 1.406 mmol) were suspended in 10 mM K,Na-phosphate buffer (10 mL), UP (238 units) and PNP (11 units) were added and reaction mixture was stirred at 40 °C for 60 h. After that time, the HPLC (Sytem A) indicated almost complete transformation of starting base into nucleoside **3b**. Precipitated guanine was filtered off; silica gel (3 mL) was added and the mixture was evaporated to dryness. The residue was twice co-evaporated with ethanol (10 mL) and put on the top of silica gel column (2 × 15 cm) that was eluted with chloroform/methanol, 15:1 (v/v). 6-Aza-2′-deoxyuridine (**3b**, 193 mg, 0.842 mmol, 90%) was obtained as colorless oil [30,75]. UV (H_2_O) λ_max_, nm (ε, M^−1^∙cm^−1^): 262 (4,000) at pH 7.0, 255 (4,300) at pH 10.0, 263 (4,000) at pH 4.0; λ_min_: 231 (1,900) at pH 7.0, 224 (2,300) at pH 10.0, 232 (1,900) at pH 4.0. Lit. data [9]: UV (phosphate buffer): λ_max_, nm (ε, M^−1^∙cm^−1^): 262 (6,900) at pH 5.0, 258 (6,900) at pH 7.0, 255 (7,200) at pH 9.0; ESIMS (positive ion mode): 252 [M + Na]^+^, 273 [M + 2Na − 2H]^+^; ESIMS (negative ion mode): 228 [M – H]^+^.

**6-Azathymidine (4b):** 6-Azathymine (100 mg, 0.787 mmol) and 2'-deoxyguanosine (315 mg, 1.18 mmol) were suspended in 10 mM K,Na-phosphate buffer (20 mL), TP (390 units) and PNP (400 units) were added and reaction mixture was stirred at 40 °C for 72 h. After that time HPLC (System A) indicated almost complete conversion of starting base into its nucleoside. Precipitated guanine was filtered off and filtrate was evaporated to dryness. The residue was dissolved in methanol, filtrated and to the filtrate silica gel (2 mL) was added. The mixture was evaporated to dryness and the residue was put on top of a silica gel column (2 × 10 cm) that was eluted with chloroform/methanol, 15:1 (v/v). 6-Azathymidine (153 mg, 0.630 mmol, 80%) was obtained as colorless oil [30,75]. UV (H_2_O) λ_max_, nm (ε, M^−1^∙cm^−1^): 263 (5,300) at pH 4.0, 263(5,300) at pH 7.0, 252 (6,000) at pH 10.0; λ_min_: 234 (2,400) at pH 4.0, 234 (2,300) at pH 7.0, 223 (3,800) at pH 10.0. Lit. data [26]: UV λ_max_, nm (ε, M^−1^∙cm^−1^): 250 (0.1 N HCl) and 265 (0.1 N NaOH); λ_min_, nm: 225 and 235. ESIMS (positive ion mode): 266 [M + Na]^+^, 282 [M + K]^+^; ESIMS (negative ion mode): 242 (M – H]^+^.

**6-Aza-2-thiothymidine (5b).** 2-Thio-6-azathymine (28 mg, 0.193 mmol) and 2′-deoxyguanosine (77 mg, 0.288 mmol) were suspended in 10 mM K,Na-phosphate buffer (15 mL), PNP (274 units) and TP (145 units) were added and the reaction mixture was stirred at 40 °C and the formation of the products was monitored by HPLC (System A). After 72 h the reaction rate significantly decreased. Precipitated guanine was filtered off; silica gel (2 mL) was added to the filtrate and solvent removed in vacuum. The residue was co-evaporated with ethanol (10 mL) and put on top of silica gel column (2 × 10 cm) that was eluted with chloroform/methanol, 30:1 (v/v). 2-Thio-6-azathymidine (**5b**) was obtained as white powder (25 mg, 0.097 mmol, 50%), that was recrystallized from acetonitrile; mp 174–176 °C.

UV spectra (H_2_O): λ_max_, nm (ε, M^−1^∙cm^−1^): 272 (18,100) and 220 (11,900) at pH 7.0; 263 (21,800) and ca. 238 (shoulder; ca. 14,000) at pH 10.0, 272 (18,000) and 219 (12,100) at pH 4.0; λ_min_: 240 (6,400) at pH 7.0, 217 (9,300) at pH 10.0, 240 (5,600) at pH 4.0.

**Figure 10:**
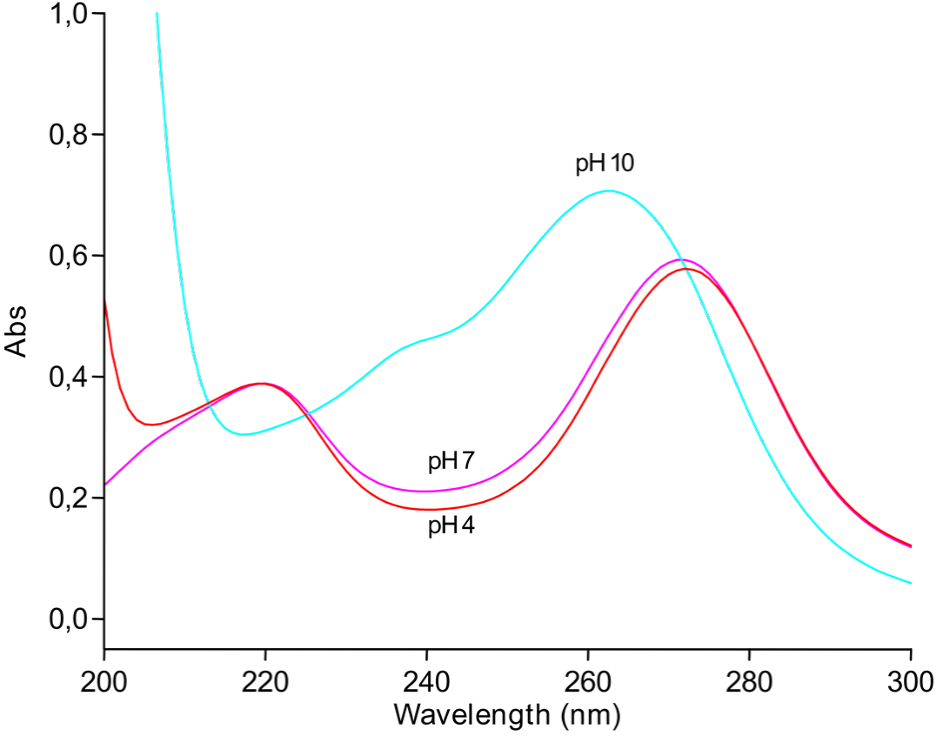
The UV spectra of 6-aza-2-thiothymidine (**5b**).

Vorbrüggen and Strelke [59] quoted the following UV data for 2-thio-6-azauridine: λ_max_, nm: (H_2_O) 269 (8,200) and 218 (12,600); (0.01 N NaOH) 267 (18,850) and 236 (12,600). ESIMS (positive ion mode): 282 [M + Na]^+^, 298 [M + K]^+^; ESIMS (negative ion mode): 258 [M – H]^−^.

## Supporting Information

File 1 Analytical and computational data.
