# Supplementary material for: Enzymatic synthesis and phosphorolysis of 4(2)-thioxo- and 6(5)-azapyrimidine nucleosides by E. coli nucleoside phosphorylases
Source: Beilstein J Org Chem. 2016 Dec 1;12:2588–601. doi: 10.3762/bjoc.12.254 (PMC5238616; doi:10.3762/bjoc.12.254)
Supplement: File 1 — Analytical and computational data. [file Beilstein_J_Org_Chem-12-2588-s001.pdf]

Supporting Information

for

Enzymatic synthesis and phosphorolysis of 4(2)-thioxo- and 6(5)-azapyrimidine nucleosides by *E. coli* nucleoside phosphorylases

Vladimir A. Stepchenko[1], Anatoly I. Miroshnikov[2], Frank Seela[3], Igor A. Mikhailopulo[1]*

Address: [1]Institute of Bioorganic Chemistry, National Academy of Sciences, Acad. Kuprevicha 5/2, 220141 Minsk, Belarus, [2]Shemyakin and Ovchinnikov Institute of Bioorganic Chemistry, Russian Academy of Sciences, Miklukho-Maklaya 16/10, 117997 GSP, Moscow B-437, Russia and [3]Laboratory of Bioorganic Chemistry and Chemical Biology, Center for Nanotechnology, Heisenbergstraße 11, D-48149 Münster, Germany

Email: Igor A. Mikhailopulo* - imikhailopulo@gmail.com.

*Corresponding author

Analytical and computational data

Table of contents

1 – **Table S1**. ^{1}H NMR spectral data for synthesized 4(2)-thio- and 6-aza-pyrimidine nucleosides **1a**–**5a**………………………….…………….........…...S2

2 – **Table S2**. ^{13}C NMR spectral data for synthesized 4(2)-thio- and 6-aza-pyrimidine nucleosides **1a**–**5a**…………………..………………………………...S3

3 – **Table S3**. The *ab initio* (6-31G** level; basis set; RHF; HyperChem 8.1) calculations of the electronic structure of 4(2)-thio- and 6-aza-pyrimidines.....S4

4 – **Table S4**. Geometry optimization using (*1*) Molecular Mechanics Force Field Bio+ [CHARMM27] and (*2*) PM3 semi-empirical method………….......S6

5 – **Table S5**. HPLC data for compounds studied...S12

6 – UV and NMR data for 6-aza-2-thio-2′-deoxythymidine...S13

7 – NMR data for 2-thio-2′-deoxyuridine...S16

1 - Table S1: ^{1}H NMR spectral data for synthesized 4(2)-thioxo- and 6-aza-pyrimidine nucleosides **1b–5b**.[a)]

Compound	^{1}H Chemical Shifts, δ_{TMS}, ppm								Coupling Constance, *J*, Hz							Remarks
	H1′	H2′	H2″	H3′	H4′	H5′	H5″	Others	1′,2′	1′,2″	2′,3′	2″,3′	3′,4′	4′,5′	4′,5″	
**1b**<br>(^{4S}U$_d$)	6.02<br>t	2.18<br>dt	2.27<br>ddd	4.26<br>dt	3.88<br>dt	3.65<br>dd	3.57<br>dd	7.54<br>(d, $J_{5,6}$ = 7.6; H6)<br>6.35 (d, H5)	**6.50**	**6.50**	**6.5**<br>^{gem}J = -14.1	**4.30**	4.15	3.50	5.10<br>^{gem}J = -<br>12.5	cf. [1]
**2b**<br>(^{2S}U$_d$)	6.81<br>t	2.16<br>ddd	2.49<br>ddd	4.31<br>dt	3.98<br>dt	3.76<br>dd	3.67<br>dt	7.91<br>(d, $J_{5,6}$ = 8.1; H6)<br>6.04 (d, H5)	6.4	6.4	6.5<br>^{gem}J = -14.2	4.3	4.1	3.4	6.5<br>^{gem}J = -<br>12.6	cf. [2-4]
**3b**<br>(6-Aza-U$_d$)	6.38<br>dd	2.21<br>ddd	2.57<br>ddd	4.48<br>dt	3.87<br>dt	3.63<br>dd	3.51<br>dd	7.47 (s, H5)	**4.7**	**7.3**	6.8<br>^{gem}J = -14.0	5.3	6.3	4.0	6.5	cf.[5,6]
**4b**<br>6-Aza-T$_d$)	6.28<br>dd	2.11<br>br.dt	2.50<br>br.dt	4.37<br>br.dt	3.79<br>m	3.57<br>dd	3.46<br>dd	2.03 (s, 3H; C^5-**CH**$_3$)	**5.0**	**7.0**	5.40<br>^{gem}J = -13.5	5.40	5.40	4.0	6.50<br>^{gem}J = -<br>12.1	Cf. [6]
**5b**<br>(6-aza-^{2S}T$_d$)	7.09<br>dd	2.18<br>ddd	2.47<br>dt	4.41<br>dt	3.83<br>dt	3.59<br>dd0	3.49<br>dd	2.08 (s, 3H; C^5-**CH**$_3$)	**4.1**	**7.2**	6.7<br>^{gem}J = -13.8	6.03<br>6.03<br>5.43	6.20<br>4.51<br>4.51	**3.90**	**6.50**<br>^{gem}J = -12.2	

[a)] The NMR spectra were measured on Bruker Avance-500-DRX (Bruker, Germany) using D_2O as a solvent if it is not stated otherwise.

References

1) Zhang, X.; Xu, Y.-Z. *Molecules* **2011**, *16*, 5655-5664.
2) Cleve, G.; Hoyer, Schulze, G., Vorbrüggen, H. *Chem. Ber*. **1973**, *106*, 3062-3072.
3) Bretner, M.; Kulikowski, T.; Dzik, J.M.; Balinska, M.; Rode, W.; Shugar, D. *J. Med. Chem.* **1993**, *36*, 3611-3617.
4) Poopeiko, N.E.; Poznanski, J.; Drabikowska, A.; Balzarini, J.; De Clercq, E.; Mikhailopulo, I.A.; Shugar, D.; Kulikowski, T. *Nucleosides & Nucleotides* **1995**, *14* (3-5), 435-437.
5) Seela, F.; Chittepu, P. *J. Org. Chem*. **2007**, *72*, 4358-4366.
6) Kögler, M.; Busson, R.; De Jonghe, S.; Rozenski, J.; Van Belle, K.; Louat, T.; Munier-Lehmann, H.; Herdewijn, P. *Chemistry & Biodiversity* **2012**, *9*, 536-556.

2 - Table S2: ^{13}C NMR spectral data for synthesized thioxo- and aza-pyrimidine nucleosides **1b–5b** [a)]

Compound	C2	C4	C5	C5-Me	C6	C1'	C2'	C3'	C4'	C5'
**1b** ($^{4S}U_d$)	149.05	190.55	113.92	-	136.92	86.16	39.13	70.6	87.03	61.09
**2b** ($^{2S}U_d$)	175.65	163.50	106.78	-	141.83	89.79 [d)]	39.14	70.07	87.19 [d)]	60.84
**3b** (6-Aza-U_d)	148.69	158.42	136.95	-	-	86.27 [d)]	36.88	71.18	86.97 [d)]	62.15
**4b** 6-Aza-T_d)	149.97	158.17	145.60	15.71	-	85.65 [d)]	36.70	71.09	86.66 [d)]	62.00
**5b** (6-aza-$^{2S}T_d$)	174.20	153.77	150.72	15.77	-	90.12	37.29	70.94	87.02	61.88

a) The NMR spectra were measured on Bruker Avance-500-DRX (Bruker, Germany) using D_2O as a solvent if it is not stated otherwise.

b) Data are in fair agreement with previously reported [1] In the transition from dU [1] to dU^{4S} (i) the C2 & C5 resonances are moved to lower field by -26.85 and -10.84 ppm, respectively, and (ii) the C2 & C6 resonances |are moved to higher field by +2.75 and +4.63 ppm, respectively.

c) Cf. [2].

d) Data for C1′ and C4′ resonances maybe interchangeable.

e) In the transition from 6-Aza-dT (cf. [4]) to 6-Aza-dT^{2S} (i) the C2 & C5 resonances are moved to lower field by -24.23 and -5.12 ppm, respectively, and (ii) the C4 resonance is moved to higher field by +4.40 ppm.

References

1 Zhang, X.; Xu, Y.-Z. *Molecules* **2011**, *16*, 5655-5664.

2 Carlucci, M.; Kierzek, E.; Olejnik, A.; Turner, D.H.; Kierzek, R. *Biochemistry* **2009**, *48*, 10882-10893.

3 Seela, F.; Chittepu, P. *J. Org. Chem*. **2007**, *72*, 4358-4366.

4 Kögler, M.; Busson, R.; De Jonghe, S.; Rozenski, J.; Van Belle, K.; Louat, T.; Munier-Lehmann, H.; Herdewijn, P. *Chemistry & Biodiversity* **2012**, *9*, 536-556.

3 - Table S3: The *ab initio* (6-31G**; basis set; RHF) calculations of the electronic structure of 2(4)-thioxo- and 6-aza-pyrimidines.

Compound	N1	N3	C^2=***O***		C^2=***S***		C^4=***O***		C5	N6	C6	Comments
	(H1)	(H3)						(C^4=***S***)	H5		H6	[Dipole moment (D)]
Uracil	-0.783	-0.830	1.020	-0.598	-	-	-0.584	-	-0.342	-	0.225	**E_T = -258 836.3 kcal/mol**
	(0.343)	(0.348)					0.182		0.192		0.197	(**4.723** D)<br>**ΔE_T = E_T – E_{T2} = -11.6**
Ura-C^4-OH	**-0.631**	-0.822	0.946	-0.599	-	-	C +0.816	-	-0.357		0.213	**E_T = -258 809.3 kcal/mol**
C^2=O		(0.330)					O -0.615<br>H +0364		0.182		0.173	(**5.758** D)
**Ura-C^4=O**	**-0.675**	-0.789	0.962	-0.612	-	-	-0.603	-	-0.357	-	0.213	**E_{T2} = -258 824.7 kcal/mol**
**C^2-OH**		(0.349)		0.382			0.794		0.182		0.173	(**3.487** D)
Ura-C^4=S	-0.797	-0.749	1.020	-0.588	-	-	-	**-0.247**	-0.281	-	0.203	**E_T = -461 291.6 kcal/mol**
C^2=O	(0.348)	(0.363)						C 0.266	0.204		0.236	(**5.672** D)<br>**ΔE_T = E_T – E_{T2} = -10.0**
Ura-C^4SH	**-0.641**	-0.754	0.944	-0.593	-	-	-	**C** 0.228	-0.324	-	0.214	**E_T = -461 269.7 kcal/mol**
C^2=O		(0.338)						**S+0.150**<br>(H +0.054)	0.180		0.173	(**5.758** D)
**Ura-C^4=S**	**-0.656**	-0.703	0.963	-0.606	-	-	-	**-0.287**	-0.261	-	0.176	**E_{T2} = -461 281.6 kcal/mol**
**C^2-OH**		(0.365)		(0.386)				0.247	0.191		0.183	(**5.171** D)
Ura-C^4=O	-0.705	-0.753	-	-		**-0.287**	-0.571	-	-0.328	-	0.217	**E_T = -461 290.1 kcal/mol**
C^2=S	(0.359)	(0.365)			0.489		0.807		0.202		0.205	(**5.322** D)<br>**ΔE_T = E_T – E_{T2} = -12.9**
Ura-C^4-OH	**-0.556**	-0.736	-	-	0.423	**-0.306**	C +0.816	-	-0.336	-	0.213	**E_T = -461 263.2 kcal/mol**
C^2=S		(0.346)					O -0.609<br>H +0369		(0.194)		0.184	(**7.561** D)
**Ura-C^4=O**	**-0.601**	-0.760	-	-	0.429	**S 0.085**	-0.602	-	-0.306	-	0.149	**E_{T2} = -461 277.2 kcal/mol**
**C^2-SH**		(0.346)				H 0.107	0.791		0.185		0.176	(**2.952** D)

**6-Aza-Ura**	-0.549	-0.826		**-0.588**	-	-	**-0.557**		-0.016	-0.184	-	**E_T = -268 849.3 kcal/mol**
	(0.356)	(0.354)	1.020		-	-	0.779		+0.211			(**1.815** D)<br>**ΔE_T** = E_T – E_T = **-18.6**
**6-Aza-Ura**	**-0.371**	-0.813	0.937	-0.572	-	-	-0.608	-	-0.095	-0.171		E_T = -268 807.9 kcal/mol
**C4-OH**		(0.339)			-	-	0.790	-	0.196			(**7.314** D) O^4-**H** 0.367
**6-Aza-Ura**	**-0.465**	-0.790		-0.608	-	-	-0.572	-	-0.012			E_T = -268 830.7 kcal/mol
**C^2-OH**		(0.355)	0.966		-	-	0.769		+0.201	-0.231		(**3.089** D) O^2-**H** 0.387

**6-Aza-Thy**	**-0.548**	-0.828		-0.595	-	-	**-0.561**	-	**0.125**	**-0.245**	-	**E_T = -293 350.2 kcal/mol**
	(0.352)	(**0.352**)	1.019		-	-	0.795	-	-		-	(**2.180** D)<br>**ΔE_T** = E_T – E_T = **-29.3**
6-Aza-Thy	**-0.370**	-0.815	0.939	**-0.578**	-	-	**-0.617**			**-0.203**	-	E_T = -293 306.7 kcal/mol
**C4-OH**		(**0.336**)			-	-	0.795				-	(**7.184** D) ^{4}O-H 0.366
6-Aza-Thy	**-0.409**	-0.827	0.965	**-0.591**			**-0.579**			**-0.288**	-	E_T = -293 320.9 kcal/mol
**C^2-OH**		(**0.334**)					0.784				-	(**4.102** D) ^{2}O-H 0.359
**6-Aza-Thy-**	**-0.469**	-0.775	-	-		**-0.277**	**-0.549**	-	**0.140**	**-0.250**	-	**E_T = -495 804.5 kcal/mol**
**C^2=S**	(0.369)	(**0.368**)	-	-	0.487		0.793	-			-	(**3.486** D) 6-31G**<br>**ΔE_T** = E_T – E_T = **-20.8**
	**-0.305**	-0.689				**-0.258**	**-0.615**	-		**-0.214**	-	E_T = -495 764.8 kcal/mol
**C^2=S; C^4-OH**		(**0.370**)			0.402		0.765	-			-	(**9.316** D) ^{4}O-H 0.387
	**-0.379**	-0.761				**S+0.173**	**-0.579**	-	0.147	**-0.299**	-	E_T = -495 783.7 kcal/mol
**C^2-SH; C^4=O**		(**0.342**)			0.409		0.791	-			-	(**3.206** D) ^{2}S-**H** 0.159

4 - Table S4: Geometry optimization using (*1*) Molecular Mechanics Force Field Bio+ [CHARMM27] and (*2*) PM3 semi-empirical method.

Substrate & Binding Mimic of Glu166 of the *E. coli* Uridine Phosphorylase (*Ec*UP) catalytic site	**Energy** (kcal/mol)<br>Total<br>Binding<br>Heat of Formation<br>Dipole moment (D)	The partial charges of selected atoms (*e*)										Hydrogen Bonds (Å) and bond lengths of the atoms involved
		N^1 (H^1)	C^4——O(S)——H			N^3——H••••**O**══C			C^2══O(S)••••**H**N			
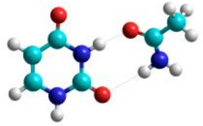	**-51,160.1**<br>-2,134.6<br>-122.7<br>DM: **3.783** D[1)]	0.083<br>(0.096)	0.294	-0.345	-	-0.046	0.179	-0.398	0.214	-0.426	0.085	**N**^{3}H•••**O**=C 2.817<br>C^2=**O**•••H**N**H 2.484
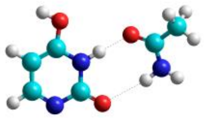	**Δ*E* = +8.9**<br>-51,151.2<br>-2,125.7<br>-113.8<br>DM: 4.120 D	**-0.202**	0.142	-0.213	0.218	0.044	0.176	-0.420	0.263	-0.386	0.090	**N**^{3}H•••**O**=C 2.816<br>C^2=**O**•••H**N**H 2.444
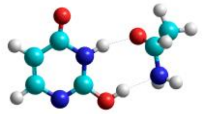	**Δ*E* = +16.1**<br>-51,144.0<br>-2,118.5<br>-106.6<br>DM: **6.595** D	**-0.214**	0.302	-0.373	-	-0.022	0.145	-0.366	0.126	-0.223	0.214	**N**^{3}H•••**O**=C 2.829<br>C^2-**O**-H•••N(H2) 2.873

[1)] The most populated tautomer is characterized by the lowest value of dipole moment (Debye).

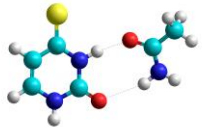	**Δ*E* = +6.5**<br>-48,654.5<br>-2,070.9<br>-52.1<br>DM: 5.560 D	0.114<br>(0.111)	-0.054	-0.205	-	0.086	0.184	-0.406	0.190	**-0.406**	0.082	N^3**H•••O**=C 1.794<br>C^2=**O•••H**N(H) 2.500<br>**N**^{3}-H••• **O**=C 2.833<br>C^2=**O•••**H-**N**(H) 3.475
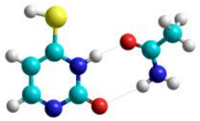 1)	**-48,661.0**<br>-2,077.4<br>-58.6<br>**DM:** **3.285** D [2]	**-0.166**	-0.130	**+0.144**<br>(S-H)	0.013	0.090	0.171	-0.422	0.247	-0.382	0.089	N^3**H•••O**=C 1.805<br>C^2=**O•••H**NH 2.436<br>**N**^{3}-H•••**O**=C 2.817<br>C^2=**O•••**H-**N** 3.358
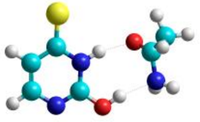	**Δ*E* = +20.6**<br>-48,640.4<br>-2,056.8<br>-38.0<br>DM: **7.583** D	-0.167	0.064	-0.270	-	0.129	0.151	-0.366	0.092	-0.215	0.222	N^3**H•••O**=C 1.848<br>C^2-O-**H•••N**(H_2) 1.923<br><br>**N**^{3}-H•••**O**=C 2.833<br>C^2-**O**-H•••**N**(H_2) 2.880

[1] Only *Ec*UP catalyzed the 2′-deoxyribosylation! (Time of reaction: 48 h; *Ec*UP & *Ec*PNP for 2-deoxy-D-ribofuranose generation; yield of dR: 39%.

[2] The most populated tautomer is characterized by the lowest value of dipole moment (Debye).

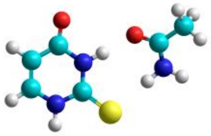	**-48,657.0**<br>-2,073.3<br>-54.6<br>DM: 2.510 D	0.197<br>(0.114)	0.272	-0.314	-	0.065	0.191	-0.416	-0.125	**-0.351**	0.111	**N**^{3}-H•••**O**=C 2.807<br>C^2=**S**•••H-**N**(H) 3.401<br>**C**^{2}=**S** 1.665<br>C^2=**S•••H**-N(H) **2.435**<br>**N**^{3}-**H** 1.025<br>N^3-**H•••O**=C 1.790
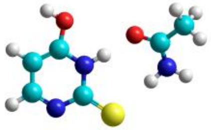	**Δ*E* ≈ +9.0**<br>-48,648.0<br>-2,064.3<br>-45.6<br>DM: 5.731 D	**-0.128**	0.105	-0.199	0.222	0.176	0.188	-0.437	-0.069	**-0.294**	0.118	**N**^{3}-H•••**O**=C 2.798<br>C^2=**S**•••H-**N(H)** 3.363<br>**C**^{2}=**S** 1.650<br>C^2=**S•••H**-N(H) **2.385**<br>**N**^{3}-**H** 1.027<br>N^3-**H•••O**=C 1.781
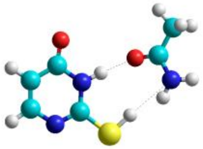	**-48,657.7**<br>-2,074.1<br>-55.3<br>DM: 5.735 D	**-0.146**	0.279	-0.369	-	0.036	0.155	-0.411	-0.126	**+0.139**<br>(H<br>0.003)	-	**N**^{3}-H•••**O**=C 2.835<br>C^{2}S-**H•••**N(H_2) 2.697<br>**C**^{2}-**S** 1.783<br>C^2-**S-H** 1.333<br>**N**^{3}-**H** 1.013<br>N^3-**H•••O**=C 1.823

2′-Deoxy-2-thiouracil (**2**b; $^{2S}U_d$) is formed in the transglycosylation reaction (Thd as a donor of 2-deoxy-D-ribofuranose; 0.4 mM phosphate buffer (pH 7.0), r.t., 24 h) in the presence of *E. coli* UP and TP in yields of 3 and 25%.

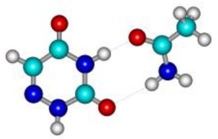	**-51,792.7**<br>- 1,999.0<br>- 97.1<br>DM: 3.430 D	**0.050**<br>(H:<br>-0.117)	0.260	-0.313	-	-0.055	0.185	-0.408	0.204	**-0.398**	0.084	**N^3**-H•••**O**=C 2.811<br>C^2=**O**•••H-**N**(H) 3.420<br>**C^2=O** 1.226<br>C^2=**O•••H**-N(H) 2.485<br>**N^3-H** 1.020;<br>N^3**-H•••O**=C 1.796<br>**N^6 -0.054 *e***
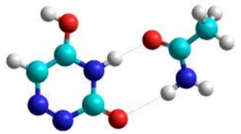	**Δ*E* = +9.6**<br>**-51,783.1**<br>- 1,989.4<br>- 87.4<br>DM: 5.617 D	**-0.136**	0.120	-0.205	0.222	0.043	0.181	-0.424	0.215	**-0.353**	0.088	**N^3**-H•••**O**=C 2.804<br>C^2=**O**•••H-**N**(H) 3.399<br>**C^2=O** 1.217<br>C^2=**O•••H**-N(H) 2.458<br>**N^3-H** 1.02<br>N^3**-H•••O**=C 1.792<br>**N^6 + 0.132 e**
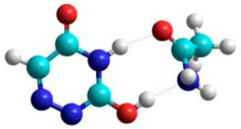	**Δ*E* = +13.8**<br>-51,778.9<br>- 1,985.3<br>- 83.3<br>DM: 8.638 D	-0.196	0.266	-0.341	-	-0.025	0.153	-0.353	0.097	-0.218	0.221	**N^3**-H•••**O**=C 2.793<br>C^2**-O**-H•••**N**(H_2) 3.576<br>**C^2-O** 1.346<br>C^2-O-**H** **N**(H_2) 2.663<br>**N^3-H** 1.013<br>N^3**-H•••O**=C 1.829<br>**N^6 +0.076 *e***

Substrate & Binding Mimic of Glu166 of the *E. coli* Uridine Phosphorylase catalytic site	**Energy** (kcal/mol)<br>Total<br>Binding<br>Heat of Formation<br>Dipole moment (D)	The partial charges of selected atoms (*e*)										Hydrogen Bonds (Å) and bond lengths of the atoms involved
		N^1<br>(H^1)	C^4—O(S)—H			N^3—H••••**O**═C			C^2═O(S)••••**H**N			
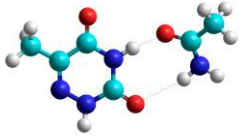	**-55,244.3**<br>-2,282.6<br>-105.5<br><br>DM = 2.994 D	0.057<br>(H 0.115)	0.267	-0.317	-	-0.054	0.184	-0.407	0.202	**-0.399**	0.084	**N**^{3}-H•••**O**=C 2.811<br>C^2=**O**•••H-**N**(H) 3.437<br>**C**^{2}=**O** 1.225<br>C^2=**O** **H**-N(H) 2.501<br>**N**^{3}-**H** 1.021<br>N^3-**H**•••**O**=C 1.804<br>**N**^{6} **-0.071 *e***
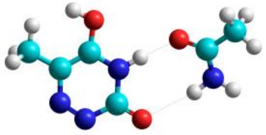	**Δ*E* = +8.7**<br>-55,235.6<br>-2,273.9<br>-96.9<br>DM = 5.374 D	-0.132	0.115	-0.211	0.227	0.046	0.181	-0.421	0.214	**-0.355**	0.086	**N**^{3}-H•••**O**=C 2.805<br>C^2=**O**•••H-**N**(H) 3.425<br>**C**^{2}=**O** 1.217<br>C^2=**O** **H**-N(H) 2.494<br>**N**^{3}-**H** 1.020<br>N^3-**H**•••**O**=C 1.790<br>**N**^{6} **+0.137 *e***
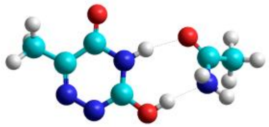	Δ*E* = +15.9<br>-55,228.4<br>-2,266.7<br>-89.7<br>DM = 8.338 D	**-0.183**	0.272	**-0.344**	-	-0.023	0.154	-0.352	0.088	0.220	N6<br>0.220	**N**^{3}-H•••**O**=C 2.841<br>C^2-**O**-H•••**N**(H_2) 2.820<br>**C**^{2}-**O** 1.341<br>C^2-O-**H** **N**(H_2) 1.862<br>**N**^{3}-**H** 1.015<br>N^3-**H**•••**O**=C 1.839<br>**N**^{6} **+0.064 *e***

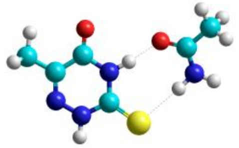	Δ*E* = +1.1<br>**-52,739.7**<br>-2,219.9<br>-36.0<br>DM = 0.523 D	0.174<br>(H 0.137)	0.245	-0.292	-	0.042	0.197	-0.421	-0.137	**-0.313**	0.112	**N**^{3}-H••• **O**=C 2.800<br>C^2=**S•••**H-**N**(H) 3.424<br>**C**^{2}**-S** 1.778<br>C^2-**S•••H**-N(H) 1.709<br>**N**^{3}**-H** 1.014<br>N^3-**H•••O**=C 1.816<br>**N^6 -0.084 *e***
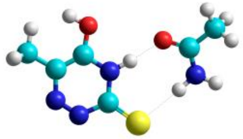	**Δ*E* = +9.7**<br>-52,731.1<br>-2,211.2<br>-27.4<br>DM = 6.413 D	**-0.064**	0.065	-0.197	0.329	0.188	0.189	-0.439	-0.136	**-0.22**7	0.115	**N**^{3}-H•••**O**=C 2.797<br>C^2=**S•••**H-**N**(H) 3.397<br>**C**^{2}=**S** 1.620<br>C^2=**S•••H**-N(H) 2.403<br>**N**^{3}**-H** 1.026<br>N^3-**H•••O**=C 1.776<br>**N^6 +0.110 *e***
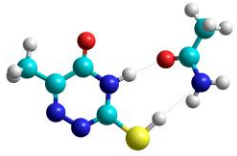	**-52,740.8**<br>-2,220.9<br>-37.1<br>DM = 7.554 D	**-0.110**	0.251	**-0.343**	-	0.037	0.162	-0.418	-0.173	**0.165**	0.002	**N**^{3}-H•••**O**=C 2.835<br>C^2-**S**-H••• **N**(H_2) 3.998<br>**C**^{2}=**S** 1.7775<br>C^2=**S-H•••**N(H_2) 1.332<br>**N**^{3}**-H** 1.013<br>N^3-**H•••O**=C 1.822<br>**N^6 +0.032 *e***

Only *Ec*TP likely recognizes the C^2-S-H ••• NH_2- tautomer as a substrate owing to (*i*) the correct positioning in the catalytic site by the interaction of methyl group with the relevant residues of amino acids, and (*ii*) higher nucleophilicity of the *N*1 atom!

5 - Table S5: HPLC data for compounds studied.

System A: Nucleosil 100-5 C_{18} 150 × 4.6, 5 µm; 5% acetonitrile in 0.1% TFA-H_2O, v/v; 1 mL/min; detection at 260 nm.
System B: Nucleodur C_{18} Gravity 150 × 4.6, 5 µm; 5% acetonitrile in 0.1% TFA-H_2O, v/v; 1 mL/min; detection at 260 nm.
System C: Nucleodur C_{18} Gravity 150 × 4.6, 5 µm; 10% acetonitrile in 0.1% TFA-H_2O, v/v; 1 mL/min; detection at 260 nm.

**Compound**	**Retention time, min**		
	**System A**	**System B**	**System C**
4-Thiouracil* (**1a**)	4.3	–	2.9
4-Thio-2′-deoxyuridine* (**1b**)	7.7	–	3.9
2-Thiouracil (**2a**)	3.3	–	2.0
2-Thio-2′-deoxyuridine (**2b**)	–	–	3.1
6-Azauracil (**3a**)	4.3	2.1	–
6-Aza-2′-deoxyuridine (**3b**)	7.7	2.9	–
6-Azathymine (**4a**)	3.7	3.6	–
6-Azathymidine (**4b**)	4.1	7.3	–
2-Thio-6-azathymine (**5a**)	5.1	–	3.3
2-Thio-6-azathymidine (**5b**)	13.4	–	4.3
2-Thiothymine	–	–	3.5
2-Thiothymidine (**11b**)	–	–	9.6
4-Thiothymine*	–	–	6.9
4-Thiothymidine* (**11a**)	–	–	8.9
Thymine	3.8	3.0	–
Thymidine	5.9	4.7	–
Uracil	–	2.0	–
2'-Deoxyuridine	–	2.6	–
Guanine	3.4	–	–
2'-Deoxyguanosine	3.4	–	–
5-Azacytosine	1.6	–	–
5-Aza-2′-deoxycytidine	5.3	–	–

*Detection at 300 nm.

6 – UV and NMR data for 6-aza-2-thio-2′-deoxythymidine

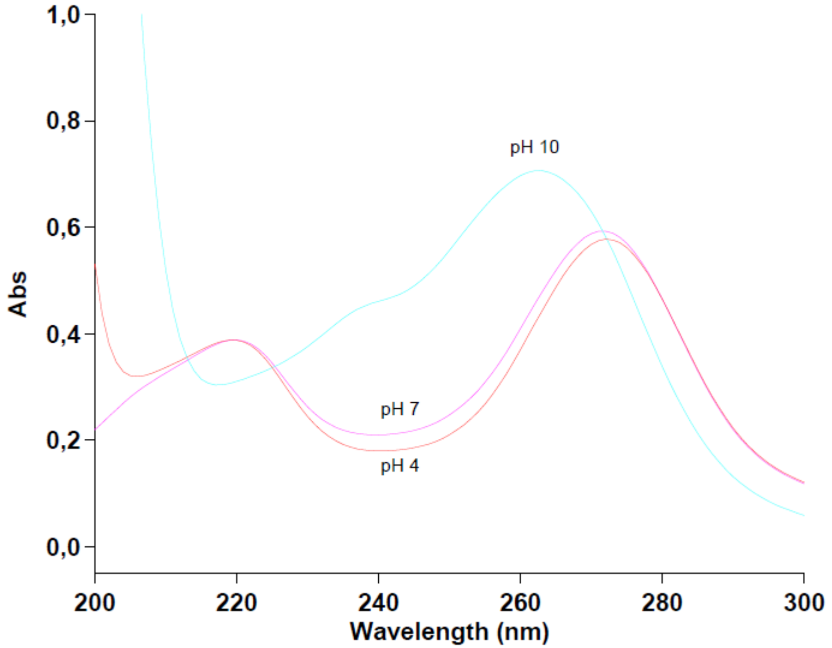

The UV spectra of 2-thio-6-azathymidine at diverse pH values.

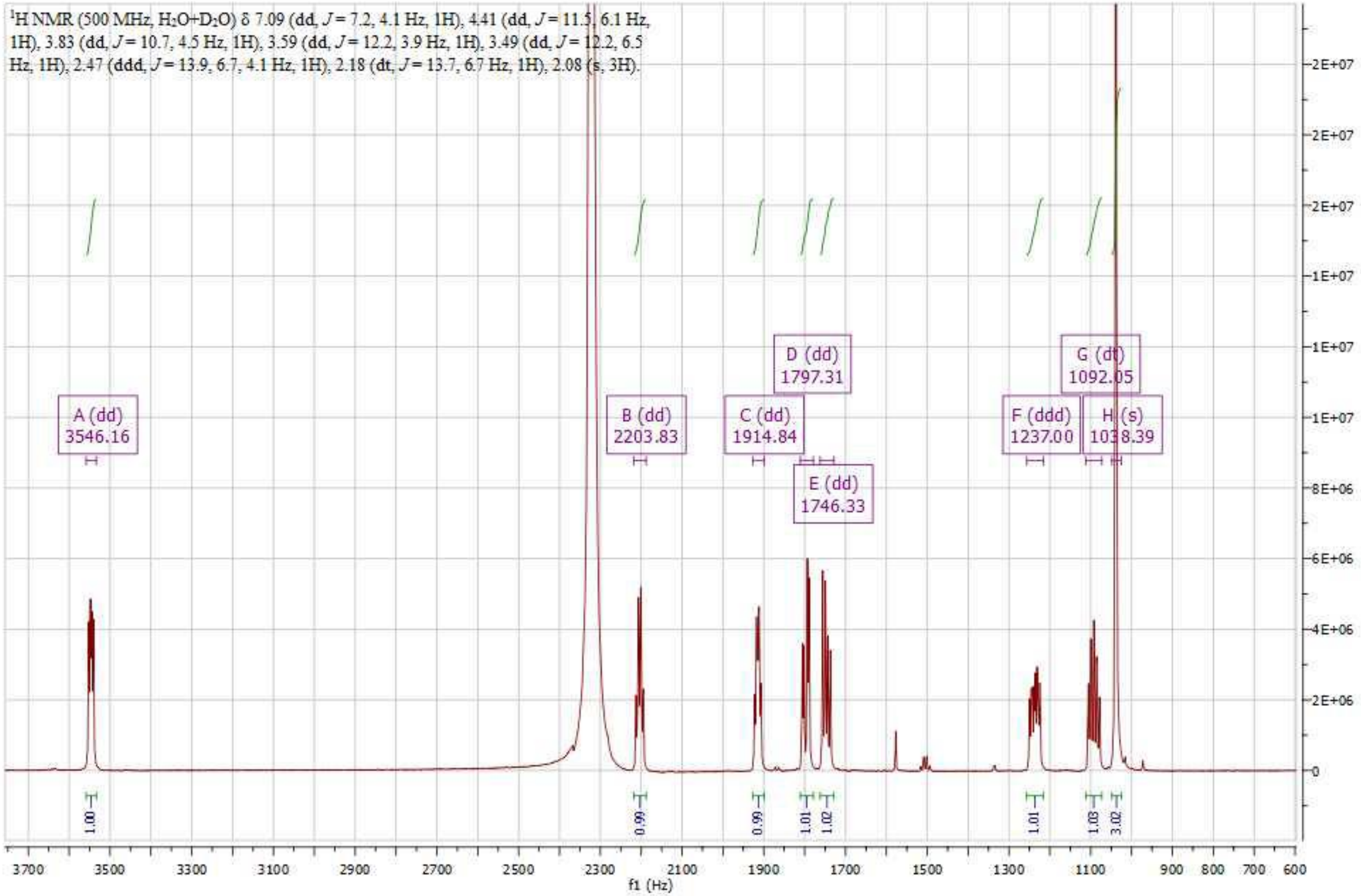

^{1}H NMR spectrum (scale in Hz)

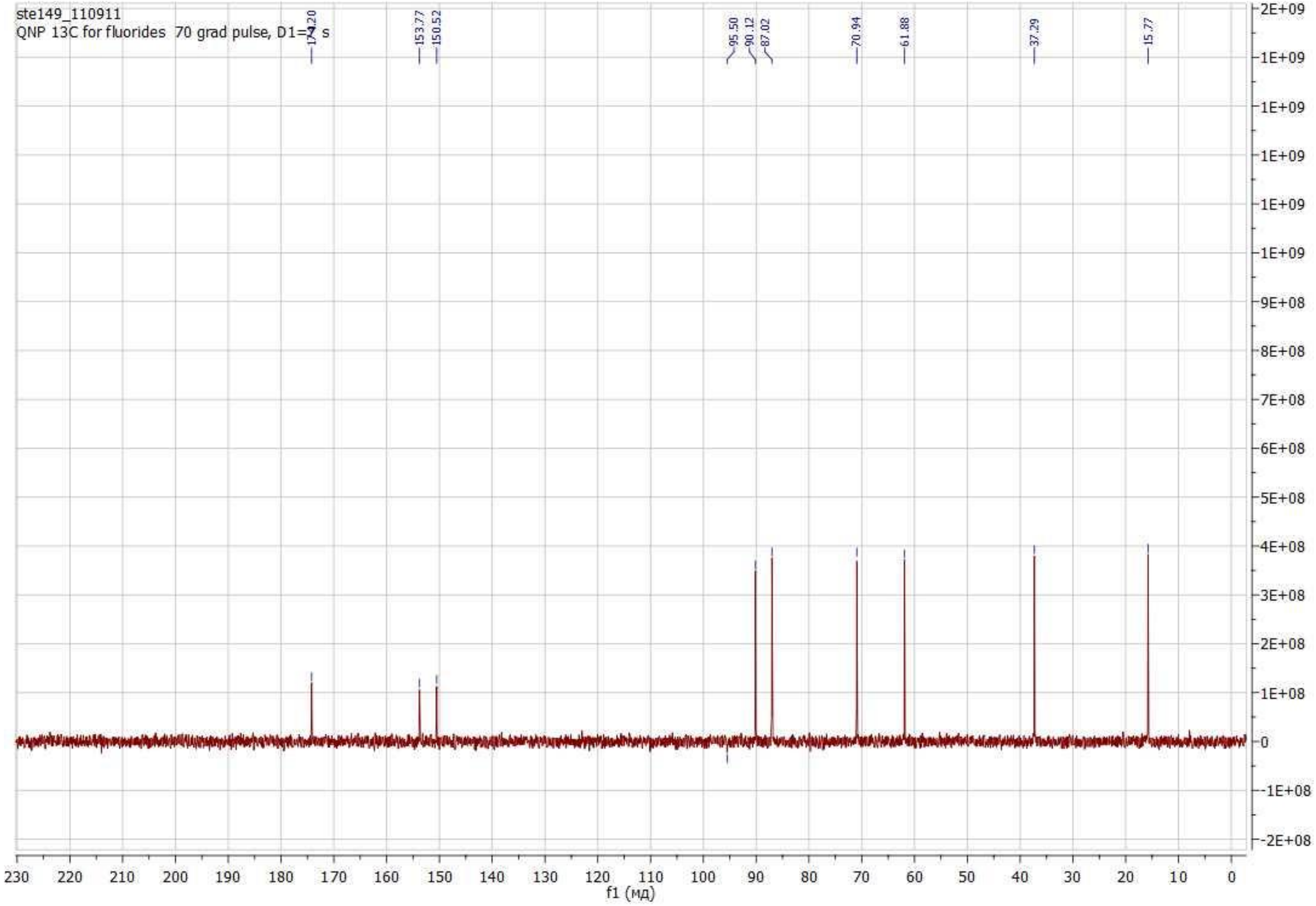

^{13}C NMR Spectrum

7 –NMR Data for 2-thio-2′-deoxyuridine

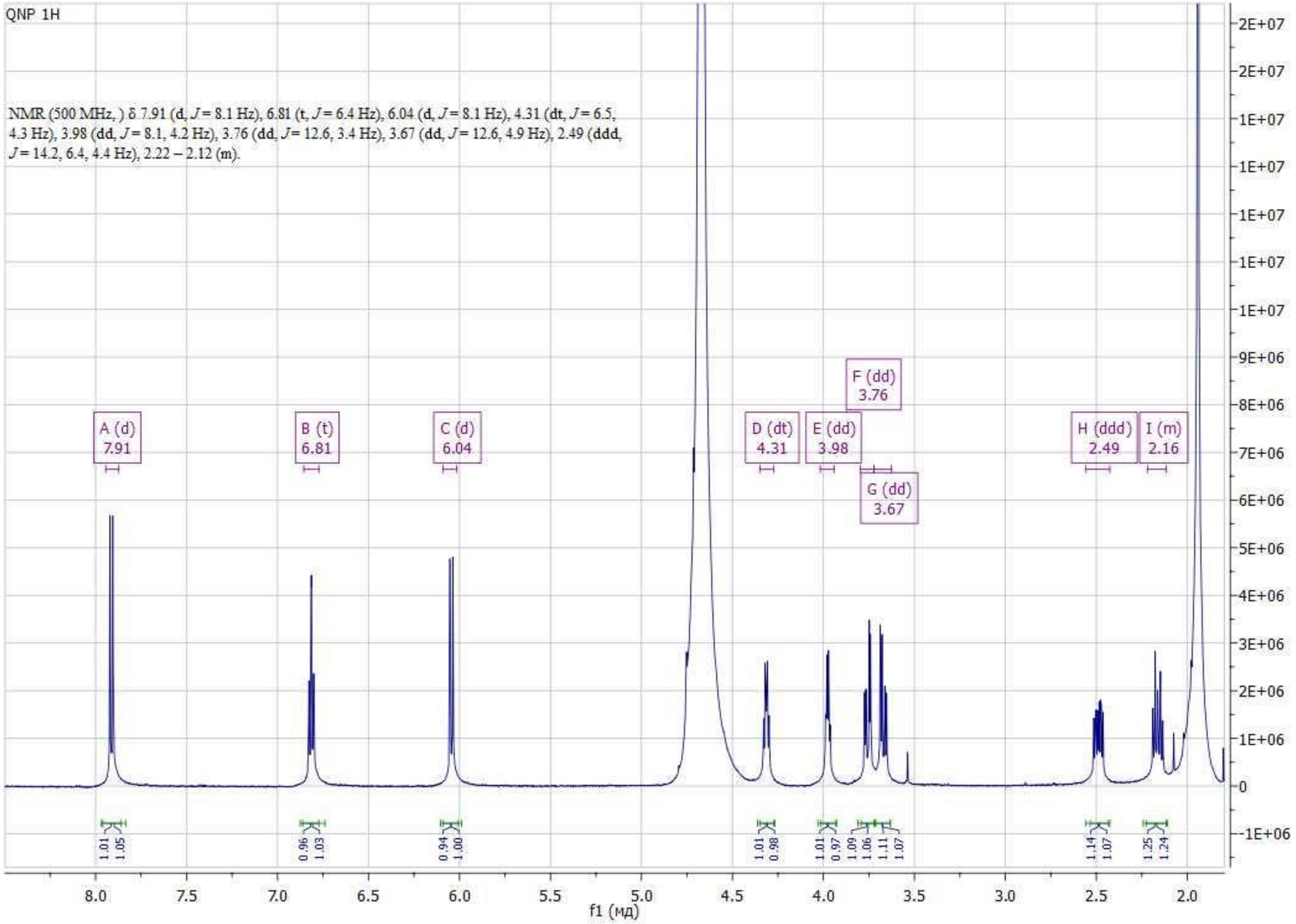

^{1}H NMR Spectrum (resonance signal of methel group of MeCN at 1.95 ppm)

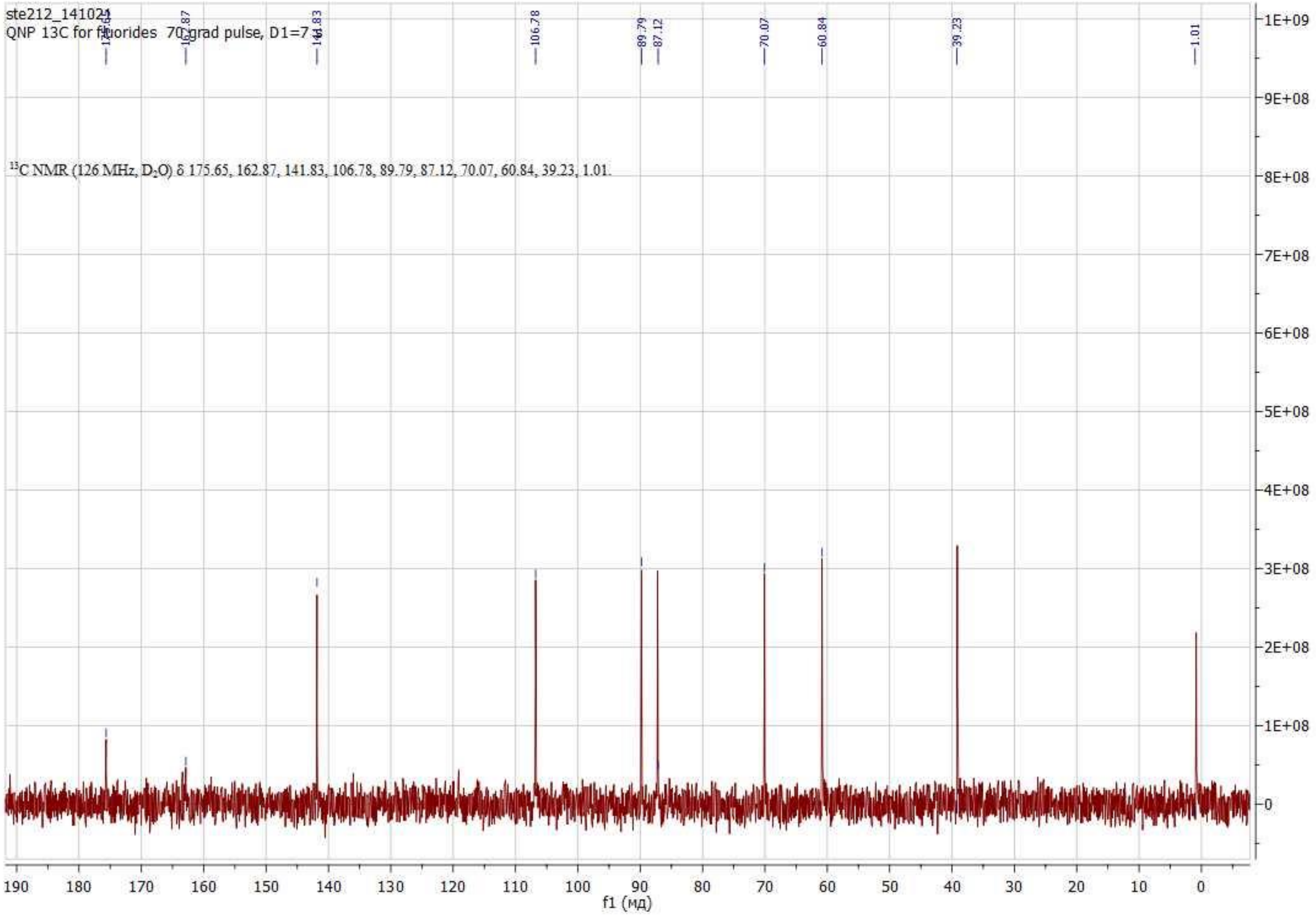

^{13}C NMR Spectrum
